# Efficient Exploration of Adsorption Space for Separations in Metal–Organic Frameworks Combining the Use of Molecular Simulations, Machine Learning, and Ideal Adsorbed Solution Theory

**DOI:** 10.1021/acs.jpcc.3c04533

**Published:** 2023-09-14

**Authors:** Xiaohan Yu, Dai Tang, Jia Yuan Chng, David S. Sholl

**Affiliations:** † School of Chemical & Biomolecular Engineering, Georgia Institute of Technology, Atlanta, Georgia 30332, United States; ‡ Oak Ridge National Laboratory, Oak Ridge, Tennessee 37830, United States

## Abstract

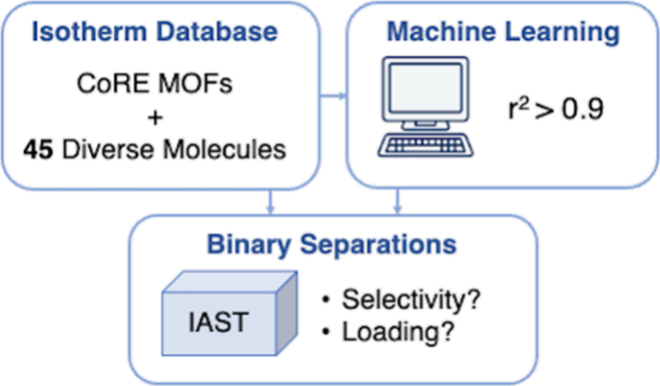

Adsorption-based separations using metal–organic frameworks (MOFs) are promising candidates for replacing common energy-intensive separation processes. The so-called adsorption space formed by the combination of billions of possible molecules and thousands of reported MOFs is vast. It is very challenging to comprehensively evaluate the performance of MOFs for chemical separation through experiments. Molecular simulations and machine learning (ML) have been widely applied to make predictions for adsorption-based separations. Previous ML approaches to these issues were typically limited to smaller molecules and often had poor accuracy in the dilute limit. To enable exploration of a wider adsorption space, we carefully selected a diverse set of 45 molecules and 335 MOFs and generated single-component isotherms of 15,075 MOF–molecule pairs by grand canonical Monte Carlo. Using this database, we successfully developed accurate (*r*^2^ > 0.9) machine learning models predicting adsorption isotherms of diverse molecules in large libraries of MOFs. With this approach, we can efficiently make predictions of large collections of MOFs for arbitrary mixture separations. By combining molecular simulation data and ML predictions with Ideal Adsorbed Solution Theory, we tested the ability of these approaches to make predictions of adsorption selectivity and loading for challenging near-azeotropic mixtures.

## 1 Introduction

Distillation and other traditional separation processes can be very energy-intensive.^1^ Adsorption-based separations are one promising, less energy-intensive alternative. Metal–organic frameworks (MOFs) make up a class of crystalline nanoporous materials with high porosity and high surface area. Their properties are highly tunable due to diverse combinations of metal centers and coordinated organic linkers. Therefore, MOFs have been widely studied for adsorption-based separations.^2^

Selectivity is critical to evaluate the performance of adsorbents. In binary mixture adsorption, the selectivity, *S*_A/B_, is defined as
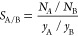
where *N*_A_ and *N*_B_ are the adsorbed loadings of each component and *y*_A_ and *y*_B_ are the mole fractions of each component in the bulk phase. While binary-component isotherms can directly provide these loadings and mole fractions, obtaining experimental mixture isotherms can be challenging.^3^ Currently, only 145 experimental binary-component isotherms for MOFs are available.^4^ Alternatively, the loadings of each component in an adsorbed mixture can be predicted from mixing models based on single-component isotherms such as Ideal Adsorbed Solution Theory (IAST).^5^ Given the broad adsorption space formed by the billions of distinct molecular species and thousands of MOFs that exist,^6,7^ even the thousands of experimental single-component isotherms that are available^8^ are not sufficient to provide a comprehensive understanding of adsorption-based separations in MOFs.

Many researchers have successfully applied computational methods, including molecular simulations and machine learning (ML), to explore the huge adsorption space defined by MOFs. A comprehensive collection of adsorption isotherms simulated by different research groups in MOFX-DB currently contains over three million data points.^9^ However, these isotherms are strongly focused on a list of small and simple molecules, including H_2_, CH_4_, CO_2_, Xe, Kr, Ar, and N_2_. This focus means that most published ML models predicting single-component or mixture adsorption properties in MOFs are only applicable to a limited collection of adsorbing species.^10−19^

One effort to broaden the range of molecules for which adsorption data in MOFs is available was made by Tang et al.^20^ In that work, adsorption isotherms of a diverse set of 24 molecules in 471 MOFs were generated by molecular simulations. Gharagheizi et al. subsequently trained ML models with this data for the task of predicting single-component adsorption isotherms of arbitrary molecules in MOFs.^21^ Examples were demonstrated of predicting selectivities of near-azeotropic molecule pairs (that is, molecules with similar bulk boiling points) from single-component isotherms combining ML-predicted single-component isotherms and IAST. It was found, however, that it is challenging to make mixture adsorption predictions without accurate descriptions of single-component isotherms in the dilute limit.^22^ To better address this issue, we recently developed a more robust ML model predicting Henry’s constants for arbitrary molecules in MOFs based on training data from a diverse collection of 45 molecules.^23^

In this paper, we present a further exploration of the adsorption space in MOFs. Single-component isotherms of a diverse set of 45 molecules in 335 MOFs at room temperature are simulated using molecular simulations. We investigate whether a universal and robust machine learning model can be trained with these data to predict single-component loadings of arbitrary molecules over the full range of adsorbed loadings possible in an MOF. We demonstrate examples of applying this ML model together with IAST to predict the selectivities of near-azeotropic pairs and discuss the limitations of this method. In addition to the potential uses of the specific ML model that we describe, we hope that the molecular simulation data described below will prompt others to tackle the full diversity of the adsorption space for MOFs.

## 2 Methods

### 2.1 Data Generation

**Figure 1:**
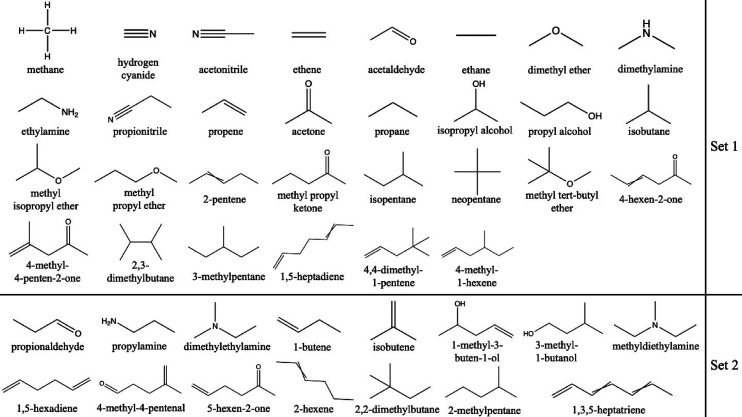
Structures of the 45 molecules used in this work.

A diverse set of 45 molecules (Figure 1) and 335 MOFs were used. This is an expansion of the work done by Tang et al.,^20^ who hand-picked 24 molecules. To better diversify the adsorbates sampled by our simulations, we selected 45 molecules that can be described by the TraPPE force field (FF)^24^ with a molecular weight <100 g/mol. Details of the molecular selection process can be found in our previous work.^23^ This collection of molecules included 13 molecules from the data set of Tang et al. and 13 near-azeotropic pairs. Information on the molecules and near-azeotropic pairs is given in Tables S1 and S2. MOF structures were obtained from the DFT-optimized CoRE MOF 2014 database.^25,26^ In our previous study, 471 MOFs were included, but only 335 of them were selected in this work to limit computational expense. Some duplicate or physically unreasonable MOFs were removed from the data set, and we further removed ∼100 MOFs because our molecular simulations for these materials failed to converge within a reasonable time with the computing resources available to us. The diversity of the MOFs remained comparable after the reduction in the number of materials (see Figure S5).

**Figure 2:**
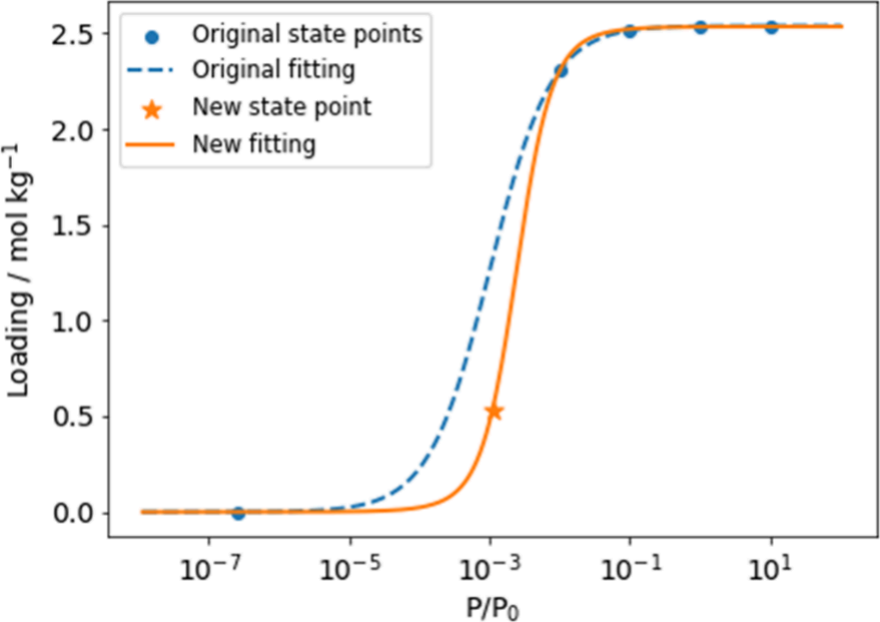
Single-component isotherm of 1-methyl-3-buten-1-ol in ABUWOJ at 300 K. Adding one state point, as indicated at intermediate loadings, allows for more reliable fitting of the overall isotherm.

A total of 15,075 single-component isotherms were simulated by grand canonical Monte Carlo (GCMC) at 300 K using RASPA.^27^ These simulations used translation, rotation, and reinsertion moves with equal probability; sample input files are available in the Supporting Information. All GCMC simulations began with an MOF containing no adsorbates. The Henry’s constant at 300 K for each MOF–molecule pair was also obtained from molecular simulations. The vapor pressure, *P*_0_, of each molecule was first predicted.^28^ We then simulated four state points in each isotherm at pressures of 0.01*P*_0_, 0.1*P*_0_, *P*_0_, and 10*P*_0_ for the molecule. We also simulated a state point in the low-pressure regime at a pressure defined to be 10^–4^ times the loading obtained at 10*P*_0_ divided by Henry’s constant of this molecule–MOF pair. This choice of pressures was made to obtain adsorbed loadings that span the full range of possible loadings for each molecule–MOF pair. Additional intermediate state points were added to some isotherms because these five pressure points did not give loadings that represented all possible conditions. Figure 2 shows an example of this situation, where adding one additional state point allows for a much more reliable description of the overall isotherm. Details of how the need for additional state points and how the pressures of additional state points were defined can be found in Section S1.1. We did not attempt to identify regions inside MOFs that may be kinetically inaccessible to adsorbing molecules even though GCMC allows insertion of the molecules in these regions.

In GCMC simulations, force field parameters for atoms in MOF frameworks were taken from the universal force field (UFF). Lennard-Jones interactions were used to model adsorbate–MOF interactions with Lorentz–Berthelot mixing rules and a cutoff of 14 Å with tail corrections. Ewald summation with a relative precision of 10^–6^ was used to calculate electrostatic interactions. We used the partial charges assigned to the MOF structures in previous work^29^ by the DDEC method.^30^ Force field parameters for molecules were taken from TraPPE-United Atom (UA) or TraPPE-Explicit Hydrogen (EH) FF.^24,31−36^ Flexible models were used to describe the molecules, including intramolecular bonds, angles, and torsions. Findley et al. showed that results using this approach were in good agreement with those using PBE-D2 DFT in a prototypical MOF when comparing CO_2_ binding energies.^37^

As is common in high-throughput treatments, MOFs were kept rigid in all calculations. We note, however, that it is known that the intrinsic flexibility of MOFs can impact adsorption even in cases where there is only a small net volume change,^38^ so assessing the potential impact of MOF flexibility is desirable in specific cases of significant interest. Our GCMC simulations used 10^5^ cycles for initialization and 10^6^ cycles for data collection. Initial tests showed that these choices gave well-converged results except in the special cases discussed below.

We took several steps to control the quality of the computed isotherms. Isotherms in rigid structures should be monotonically increasing as pressure increases, so 129 isotherms that showed a >5% relative decrease in two consecutive loadings were marked as “Decreasing”. This typically occurred due to large uncertainty in the GCMC simulations of one or more state points. The uncertainty of a GCMC simulation is computed by dividing the simulation into five blocks and calculating the resulting standard derivation. In 1,109 additional isotherms, numerical uncertainties of one or more state points were found to be larger than the loading, so these cases were marked as “Large Uncertainty”. Examples of these two types of isotherms are shown in Section S1.2. The “Decreasing” and “Large Uncertainty” isotherms were not used in subsequent model development. We marked another 2,218 isotherms as “No Adsorption” because the maximum loading in the isotherm is <10^–5^ mol/kg. The database can be accessed in the SI and through https://github.com/tdytjd/mof_diverse_isotherm_prediction.

### 2.2 Descriptors

Typical MOF descriptors used in ML adsorption predictions are geometrical descriptors,^39^ and an increasing number of studies are incorporating energy-based descriptors.^15,17^ We combined these two categories of MOF descriptors. The three geometrical descriptors we used are pore limiting diameter (PLD), largest cavity diameter (LCD), and void fraction, with values calculated by Zeo++.^40^ Potential energy surface (PES) descriptors were taken from our previous study predicting Henry’s constants.^23^ The Henry’s constants of each MOF–molecule pair were simulated in the previous work, and their log values were used as a descriptor. These descriptors do not capture the possible shapes of isotherms, which motivated us to include a Langmuir descriptor. The Langmuir adsorption model can be described as
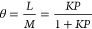
where θ is the fractional occupancy of the adsorption sites, *L* is the loading, *M* is the monolayer loading covering all adsorption sites, *P* is the pressure, and *K* is the equilibrium constant. We approximated *K* by using Henry’s constant, and θ was used as a descriptor. By doing so, we were able to make an initial physically based estimation of the isotherm shape.

We used seven more molecular descriptors to characterize adsorbates. The critical temperature, critical pressure, and acentric factor were predicted by the model developed by Gharagheizi et al.^41^ xlogP, a descriptor describing the partition coefficient of the molecule, was also used.^42^ We calculated the three diameters of the molecules using the minimum enclosed ellipsoid method^43^ and used them as descriptors for each molecule (see Section S1.3 in the Supporting Information). The complete list of descriptors can be found in Table S3.

### 2.3 Machine Learning Models

The data set was split the same way as in our previous work.^23^ Set 1 consists of data from only 30 molecules, and our models were trained on only Set 1. The remaining 15 molecules in Set 2 were not used for any model training. We first trained a classification model using a support vector classifier^44^ to decide whether adsorption is possible at any physically significant level (i.e., the maximum observed loading is >10^–5^ mol/kg) in a given MOF–molecule pair. We removed all isotherms classified as having no adsorption by the classifier from further model development. We then developed an ensemble of 10 regression models for predicting single-component isotherm loadings on the remaining isotherms using a XGBoost regressor with least-squares error as the loss function. Set 1 was split 80/20 for training/test. Hyperparameters were trained by using cross-validation. We used the scikit-learn^45^ package and XGBoost package^46^ to develop the models. Figure S6 shows a workflow for the algorithms used in the model training and calculation. All codes and models can be accessed at https://github.com/tdytjd/mof_diverse_isotherm_prediction.

### 2.4 Calculation of Binary Loadings and Selectivities

Predictions of mixture adsorption were made from single-component isotherms by using IAST. Applying IAST requires information about the single-component isotherms at arbitrary pressures to allow accurate integration of the isotherm. Although an ML-predicted isotherm can be used for this purpose, we found that it is considerably more reliable to fit a physically motivated smooth isotherm to a set of state points representing the full range of pore loadings. We used pyIAST^200^ to fit the GCMC-simulated and ML-predicted state points of an MOF–adsorbate pair to continuous curves. The ML-predicted state points were generated at the same pressures as those of the GCMC-simulated state points. We considered five adsorption models available in pyIAST, namely, the Langmuir, Quadratic, BET, Dual-site Langmuir, and Temkin isotherms. The model with the least root mean square error (RMSE) was chosen. We modified the fitting process to give more accurate results in the low-pressure regime, which is essential when using IAST with the aim of making quantitative predictions about adsorption selectivity.^22^ The details of this modification can be found in Section S1.4. We used the resulting fitted curves as the single-component isotherms for predicting binary adsorption properties with IAST.

In applying IAST to some situations, extrapolation of one or both single-component isotherms beyond the range of the original data is required. We used the default method of pyIAST, which assumes that the saturation loading is the loading calculated by the fitted isotherm equation at the highest-pressure state point. This approach can be problematic if the source data is experimental data,^47^ but it is reasonable for our calculations because our state points were selected to include the full range of possible single-component loadings.

All binary mixtures were assumed to be equimolar, unless stated otherwise. Loadings and selectivities were calculated at *P*_total_ = 0.5 × (*P*_vp,1_ + *P*_vp,2_), where *P*_vp,1_ and *P*_vp,2_ are the predicted vapor pressure of each component.^28^

## 3 Results

### 3.1 Classification of Isotherms to Remove Nonadsorbing Cases

To improve the reliability of machine learning regression models predicting adsorption properties, classification models have been used previously to predict where regression models will make reliable predictions.^21,23^ In this data set, some GCMC-simulated isotherms show extremely small or zero loadings at all state points, indicating that the MOF is effectively nonporous with respect to a specific molecule. It is challenging to decide whether the MOF is nonporous to an adsorbate by only computing the pore size and the molecule size when these two quantities are similar (Figure S7). We developed a classifier to address this issue and exclude isotherms with extremely small loadings before training regression models. This approach avoids complications of regression being biased by numerically extreme values associated with physically uninteresting cases. If the maximum loading of an isotherm for a given molecule–MOF pair is less than 10^–5^ mol/kg, the isotherm is labeled as “No Adsorption”; otherwise, it is labeled as “Non-negligible Adsorption”. Our classifier was developed to distinguish between these two classes. All isotherms predicted to be “No Adsorption” by the classifier were excluded from the regressor training described below.

Seven descriptors were used to train our support vector classifier: three MOF descriptors (PLD, LCD, and void fraction), three molecule descriptors (critical temperature (*T*_c_), critical pressure (P_c_), and acentric factor), and the log of Henry’s constant of the MOF–adsorbate pair. The code of the classifier implementation is available at https://github.com/tdytjd/mof_diverse_isotherm_prediction/Classification.

Out of 9,362 valid isotherms in Set 1, 1,365 were labeled as “No Adsorption” by GCMC simulated results. We developed a classifier with 0.999 accuracy, so fewer than ten isotherms were misclassified (Table 1). The transferability of this classifier was tested on Set 2. Predictions were made on 4,476 isotherms, with 853 of them showing no adsorption using GCMC simulation results. Only one isotherm of the unseen molecules was misclassified, which indicates that the classifier is promising for broader use. It is not surprising that a highly reliable classifier for this purpose can be defined since the qualitative question of whether a specific molecule can fit inside a rigid MOF is a relatively simple one.

**Table 1:** Classification Confusion Matrix^a^

Set 1			Set 2		
	GCMC N	GCMC P		GCMC N	GCMC P
SVC N	1,362	3	SVC N	852	1
SVC P	1	7,996	SVC P	0	3,623

a P: Non-negligible adsorption, N: No adsorption.

### 3.2 Regression Model to Predict Single Component at 300 K

Before training the regressor, we removed all isotherms predicted to be “No adsorption” by the classifier defined above (all isotherms marked as “Large Uncertainty” or “Decreasing” were already removed before training the classifier). After this step, Set 1 consisted of 43,846 distinct state points of isotherm loadings, and we used a random split of 64/16/20 training/validation/test on the loadings of each molecule. This choice means that different points in the same isotherm may belong to different splits. To better understand the uncertainties of the predictions, we created an ensemble of 10 boosting regressors. The average and standard deviation of the 10 predictions for each data point were recorded and further used for the binary adsorption calculations in Section 3.3. Each regressor was trained to minimize the mean square error with a different data split. The hyperparameters tuned by the grid search are listed in Table S4. The code implementing the model is available at https://github.com/tdytjd/mof_diverse_isotherm_prediction/regression.

The predicted loadings under different pressures ranged over many orders of magnitude. If we make predictions based purely on the loadings or, alternatively, on the log of the loadings, the model could be biased toward either higher or lower loadings. To make balanced predictions over a broad range of loadings, we applied a scaling method to the loadings. Specifically, if the GCMC loading was less than 0.1 mol/kg, then the target value was the log of the loading. GCMC loadings that were zero were assigned a log value of −10, which was smaller than the smallest nonzero loading of the whole data set. The original loading value was used as the target value if the GCMC loading was larger than 0.1 mol/kg. Our ensemble of regression models was developed using these scaled data. Distributions of the loadings before and after scaling are shown in Figure S8.

**Figure 3:**
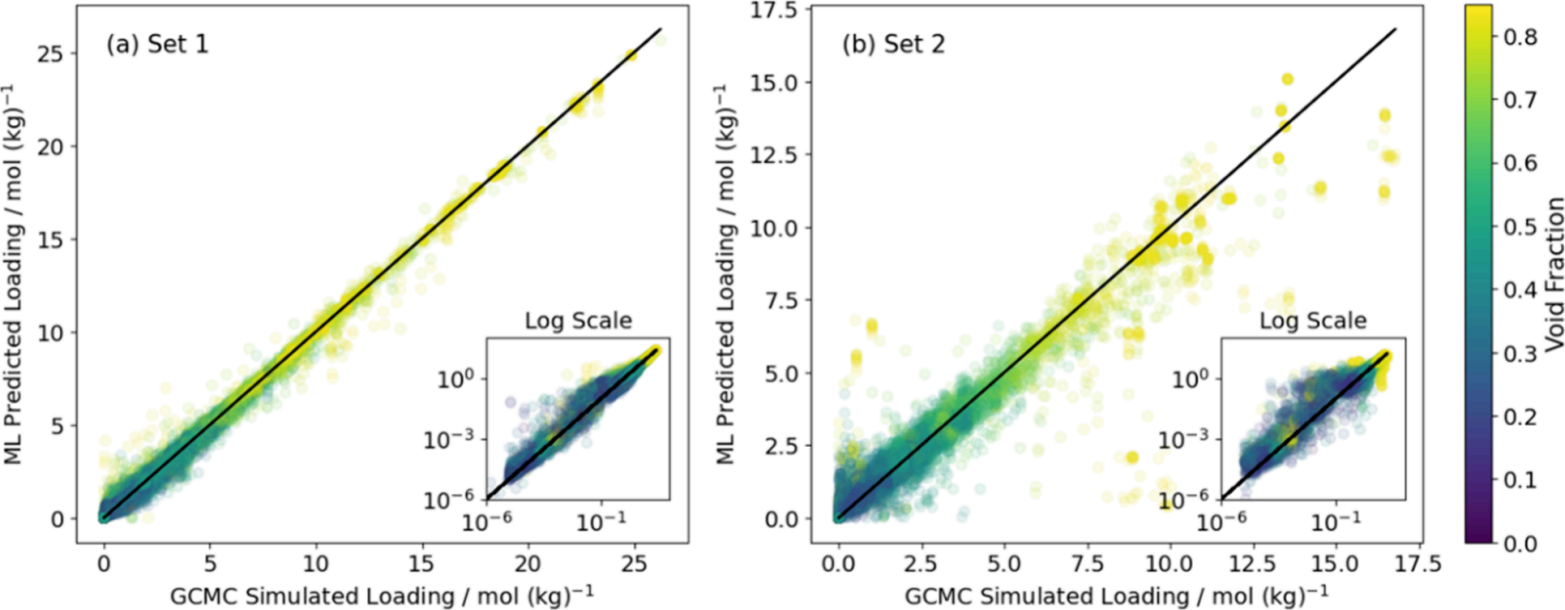
Parity plots of average ML loading predictions vs GCMC simulated loadings of (a) Set 1 and (b) Set 2. The color bar shows the void fraction of the MOFs.

**Figure 4:**
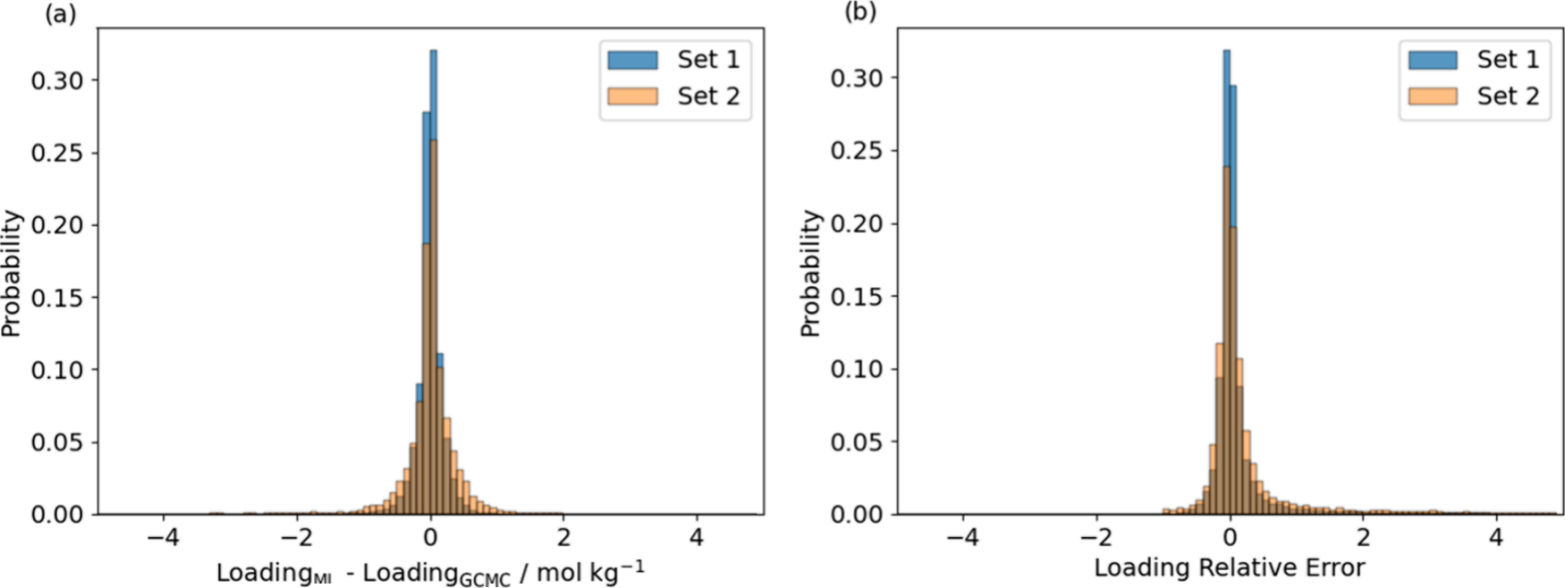
Absolute and relative error distribution of loading predictions in Set 1 and Set 2.

As shown in Figure 3, we successfully developed robust ML models with good agreement between predicted loadings and simulated loadings on both the log and normal scale. We used the coefficient of determination (*r*^2^) and mean absolute error (MAE) as the evaluation metrics, and the results are listed in Table 2. The distribution of absolute errors of predictions generally follows a normal distribution (Figure 4a), with >95% of the predictions showing a deviation smaller than 0.5 mol/kg. The relative errors of the loading predictions also follow a normal distribution (Figure 4b), with >80% of the predictions showing a relative error smaller than 0.25. Results and figures of each regressor in the ensemble can be found in Figure S9.

**Table 2:** *r*^2^ and MAE of the regression

	training	validation	test	Set 2	Gharagheizi et al.^a^
*r*^2^	0.995 ± 0.001	0.995 ± 0.002	0.973 ± 0.002	0.908 ± 0.007	0.824
MAE (mol/kg)	0.120 ± 0.013	0.120 ± 0.014	0.208 ± 0.004	0.308 ± 0.010	2.157
N^b^	28,060	7,016	8,770	19,216	42,319

a Overall results of training, validation, and test sets by Gharagheizi et al.^21^

b Number of data points.

We evaluated the transferability of our model using Set 2, which consists of 19,216 loading points from 15 molecules in 335 MOFs that were not part of the training data. The performance was still satisfactory, and the parity plots of Set 2 predictions are shown in Figure 3b. Compared with predictions on Set 1, the ensemble of models performed slightly worse on Set 2, with a smaller *r*^2^ of 0.908 and a larger MAE of 0.308 mol/kg. In the distribution of absolute errors of Set 2, the 95% confidence interval climbs to ∼1 mol/kg (see Figure 4a). More than 80% of the predictions on Set 2 show a relative error of less than 0.5, which is also slightly worse (Figure 4b). Gharagheizi et al. developed an ML model using a previously generated data set of 24 molecules in 471 MOFs.^21^ Although there was only some overlap between the lists of molecules of interest, our model is still much more accurate than this previous model for unseen molecules in terms of *r*^2^ and MAE (Table 2). Our model showed worse performance on MOFs with very large void fractions. This is evident from the outliers observed in Figure 3b, which are primarily MOFs with void fractions larger than 0.8.

**Figure 5:**
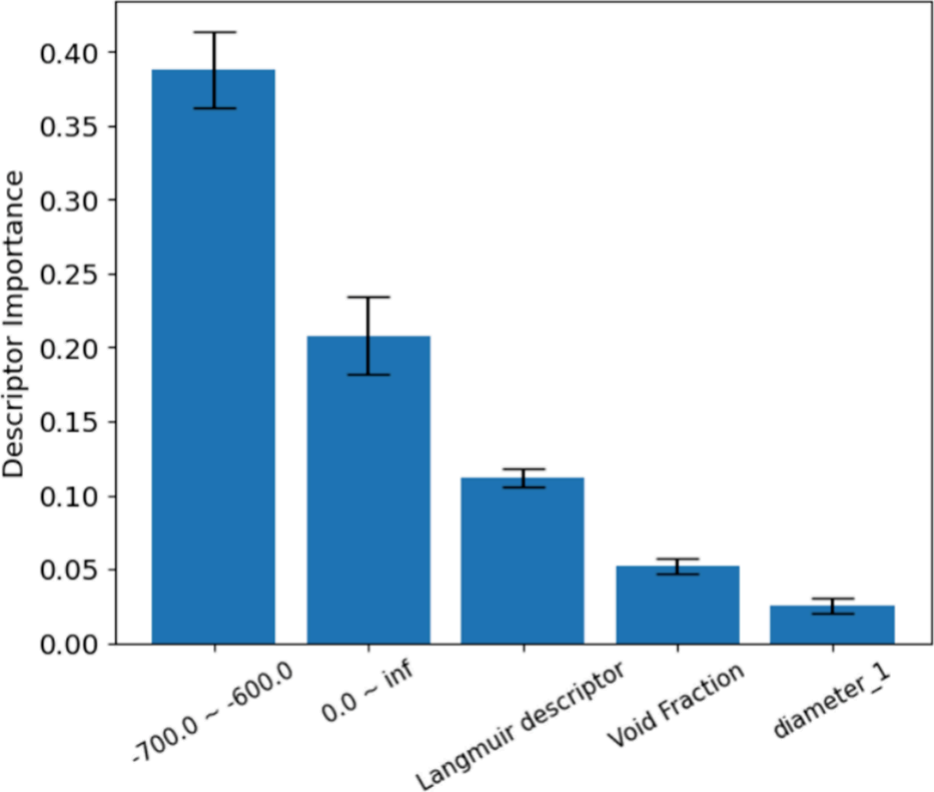
Five most important descriptors of the ML regressor and their importance.

The importance of all 41 descriptors can be found in Table S5, and the five most important descriptors are indicated in Figure 5. These five descriptors show a combination of the properties of the MOF, the properties of the adsorbate, and the properties of the isotherm. The first two descriptors from the PES category and void fraction are MOF descriptors. ‘diameter_1’ is one of the spherical descriptors for the molecule. The Langmuir descriptor is the third important descriptor, validating the importance of describing the isotherm shape. We also trained the models without the Langmuir descriptor and found that the MAE for Set 2 increased from 0.308 to 0.321 mol/kg. For some MOF–adsorbate pairs, the predictions made from the models trained without the Langmuir descriptor were significantly worse (Figure S10).

### 3.3 Binary Adsorption Predictions

IAST has been widely applied to calculate binary-component adsorption loadings and selectivities from single-component isotherms. The simulated single-component isotherms and our ML models for predicting single-component loadings made it possible for us to make useful predictions for binary-component adsorption. We have deliberately included 13 near-azeotropic pairs (10 in Set 1, 3 in Set 2) in our data set to test whether quantitative binary adsorption predictions of these challenging pairs can be made.

To gain a comprehensive assessment of IAST-predicted binary-component adsorption, we must consider the influence of the data uncertainties. Just like experimental measurements of loadings, both simulations and ML predictions have inherent uncertainties. Incorporating the uncertainties of single-component isotherm predictions into our calculations gives us insights into how trustworthy our binary adsorption calculations are. We applied the method of Gharagheizi et al. to calculate the uncertainties associated with IAST predictions made using GCMC-simulated isotherms.^47^ The uncertainty of each single-component state point was assumed to be normally distributed around the average loading. The mean and standard deviation of this distribution were taken from the reported average and 95% confidence interval in RASPA. We then randomly drew values from the distributions for all state points in the isotherm and generated 10 independent fitted isotherms for each MOF–molecule pair. When calculating the binary adsorption using GCMC-simulated isotherms, we performed IAST calculations using pyIAST on all 100 possible combinations from the two sets of 10 isotherms.

We adapted similar ideas to estimate the uncertainty in the IAST predictions associated with ML-predicted single-component isotherms. Specifically, for each MOF–molecule pair, we generated a set of 10 independent single-component isotherms from an ensemble of 10 regressors. IAST calculations were then performed for the 100 combinations of single-component isotherms for each binary mixture. This approach is successful for qualitatively evaluating MOFs’ performance on the separation of nonazeotropic pairs; an example is shown in Figure S11. We applied this approach to all of the near-azeotropic pairs of molecules in our data set.

**Figure 6:**
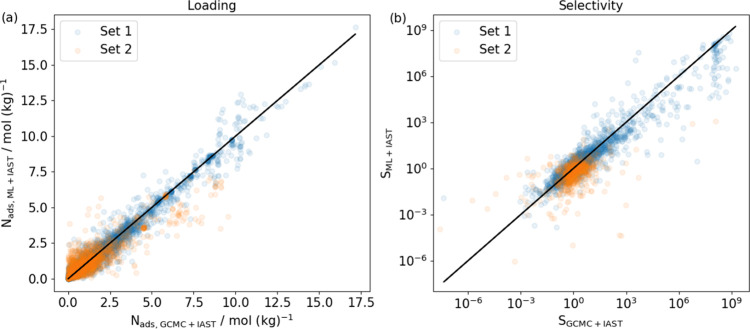
Parity plots of binary adsorption calculations from GCMC + IAST vs ML + IAST of all 13 near-azeotropic pairs in 335 MOFs at 300 K showing (a) loadings of both components and (b) adsorption selectivities.

**Figure 7:**
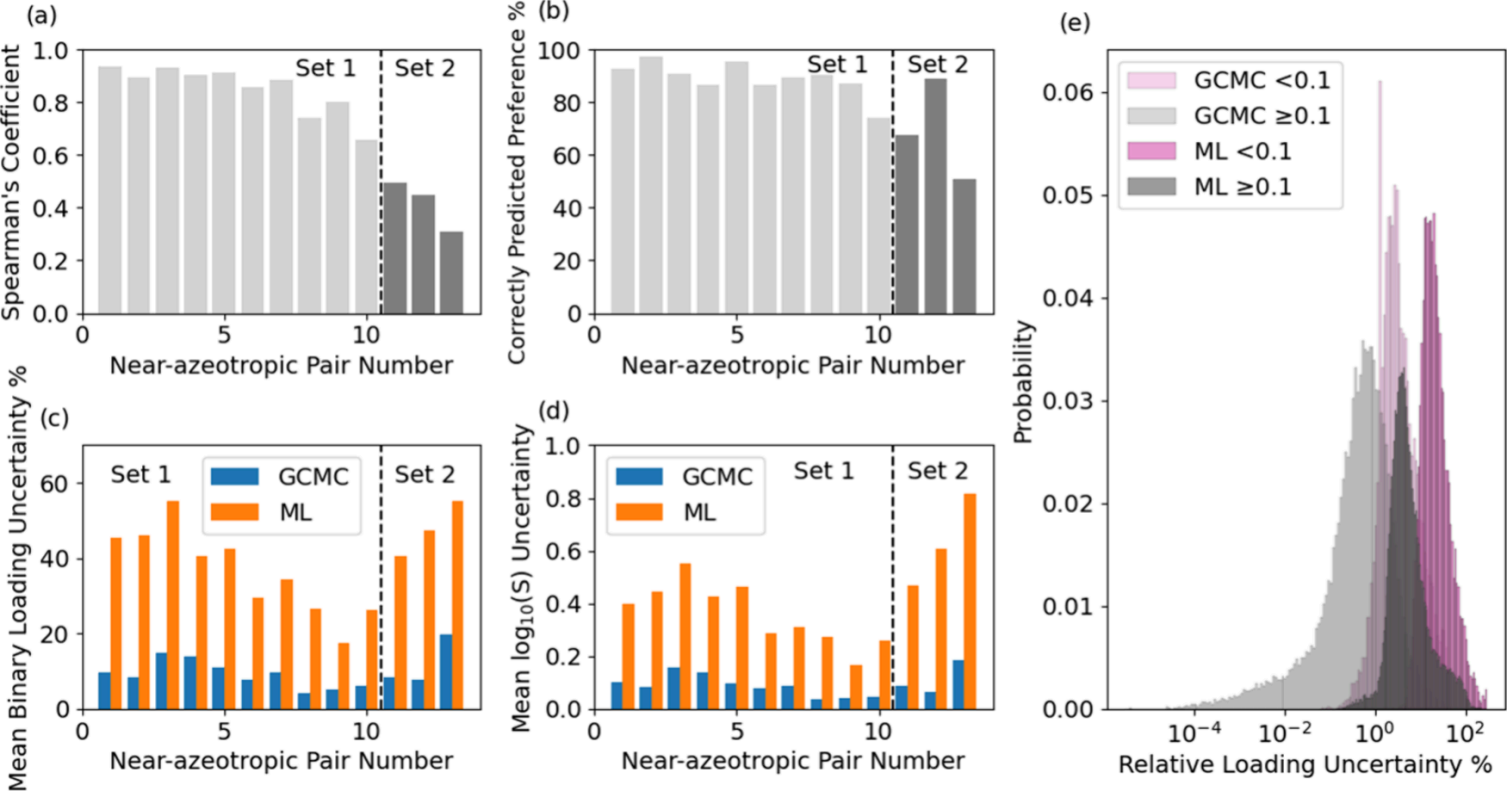
(a) Spearman’s coefficients and (b) percentage of correctly predicted preference of the 13 near-azeotropic pairs. Mean binary (c) loading and (d) selectivity uncertainties of the 13 near-azeotropic pairs. (e) Relative loading uncertainty distribution of single-component isotherms.

We performed 435,000 equimolar binary adsorption calculations on 13 near-azeotropic pairs in 335 MOFs. The overall parity plots of binary loadings and selectivities calculated from GCMC-simulated and ML-predicted single-component isotherms, respectively, are shown in Figure 6. We reiterate that in both cases, we used IAST to make predictions about mixture adsorption. Notably, there are many examples where the adsorption selectivity of near-azeotropic pairs differs strongly from the unselective (S∼1) situation that would result if adsorption of both molecules in an MOF was very similar. Parity plots with uncertainties of each near-azeotropic pair can be found in Figure S12. For each near-azeotropic pair, Spearman’s coefficient for the rank of the MOFs’ selectivities is shown in Figure 7a. We also calculated the percentage of correctly predicted preferences of each binary pair on all MOFs (Figure 7b). For most of the pairs, the correct predicted preference is above 90%. These results indicate that reasonable predictions can be made with our ML-predicted isotherms compared to those using GCMC-simulated single-component isotherms. Compared to the binary mixtures in Set 1, the three unseen mixtures in Set 2 have a worse consistency.

Most work to date using ML predicting MOF-related properties lack information on uncertainties of point predictions.^48^ By considering aspects of uncertainty here, we aim to bring attention to the value of understanding how these factors influence the interpretation of ML predictions. The mean uncertainties observed in our binary calculations are shown in Figure 7c,d. The binary predictions made using ML-predicted single-component isotherms are much more uncertain than those using GCMC-simulated isotherms because the uncertainties of ML predictions on single-component loadings are roughly 1 order of magnitude higher than those of GCMC simulations (Figure 7e).

Propane and propene are both important gases used in various applications. However, they have very similar chemical properties, and separating them can be a challenging and energy-intensive process. Many studies have reported successful propane/propene separations using a handful of MOFs by experiments,^49−51^ but it would be useful to enlarge this list of MOFs. Here, we use the separation of propene over propane (near-azeotropic pair number 9 in the results above) as an example to show how to apply the general-purpose models that we have developed to select promising MOFs rapidly for a given separation task.

**Figure 8:**
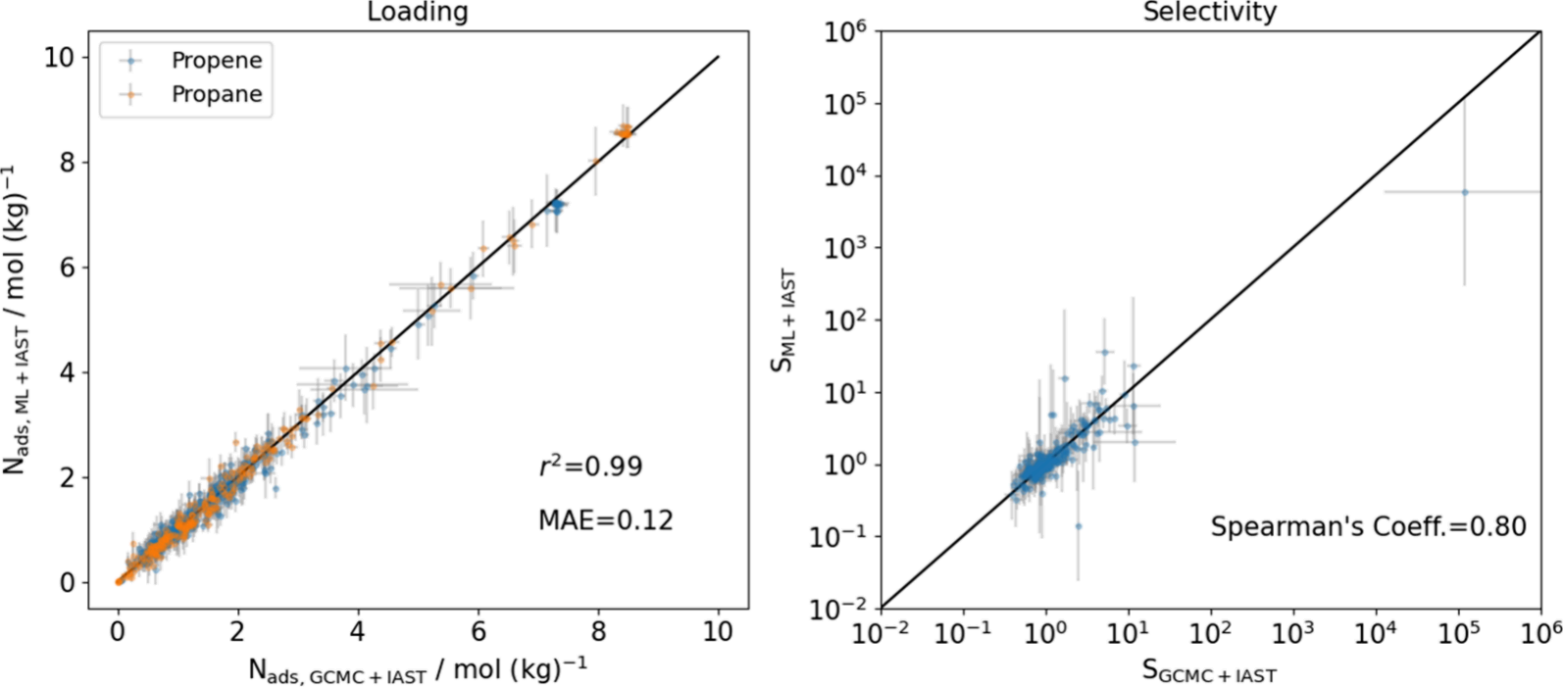
Parity plot of propene/propane separation predictions in 335 MOFs at 300 K. (a) Binary loadings and (b) selectivities.

The predictions for binary adsorption loadings and selectivities using GCMC-simulated and ML-predicted single-component isotherms for propane and propene show good agreement (Figure 8). We ranked the 335 MOFs in terms of their predicted selectivity for propene/propane separations at high loadings by using the same methods as above. The Spearman’s coefficient of 0.80 for these two methods indicates a strong similarity in the way that MOFs are ranked with the two methods. The best candidates for this separation proposed by the two methods match well. Out of the 15 top-performing MOFs identified by both methods, 10 materials were found to overlap (see Table S6). The model found four MOFs with a predicted selectivity >10 but none with a predicted selectivity <0.1 at the specific state point used for this assessment.

These example calculations for propane/propene mixtures illustrate both the strengths and weaknesses of this ML-based approach. Our ML model was trained on isotherms simulated by a generic FF in MOF structures that were assumed to be rigid. To pursue the highly ranked MOFs in more detail, it would be important to understand the potential impact of inherent flexibility of the MOFs, which is possible at an FF level,^38^ and also to test whether the FF underlying our calculations is in good agreement with higher level methods^37,52,53^ (e.g., DFT). Because the candidates that are predicted to show strong selectivity for propane/propene using FF calculations with rigid structures are likely to function based on size exclusion, the effects just mentioned may be more influential than those in MOFs where both species of interest readily fit in the pores. Our ranking was based on the predicted selectivity at a single bulk pressure corresponding to high pore loadings. A strength of our ML approach is that it can rapidly make predictions for a wide range of conditions, as illustrated in Figure S13. In each of the four examples in Figure S13, the predicted selectivity at high loadings in the MOF is very different from the selectivity in the limit of dilute adsorption.

Propane and propene are examples of a near-azeotropic pair of molecules. Tang et al. studied a very challenging adsorption-based separation task where detailed molecular simulation data was generated for 6 MOFs and 12 near-azeotropic molecules with predicted boiling points between 351.7 and 356.1 K.^22^ The Henry’s constants and single-component isotherms of the 72 MOF–adsorbate pairs at 300 K were simulated using GCMC, together with the binary-component equimolar adsorption of all 66 possible near-azeotropic molecule combinations in each MOF at 300 K and 0.5 × (*P*_vp,1_ + *P*_vp,2_). These calculations showed that IAST was applicable to this task by comparing selectivities and loadings simulated in binary-component adsorption and calculated from GCMC-simulated single-component isotherms using IAST. They attempted to make analogous predictions using an ML approach, but the binary adsorption properties calculated using ML-predicted single-component isotherms with IAST were inaccurate because of poor predictions by their ML model for low and moderate pressures.

**Figure 9:**
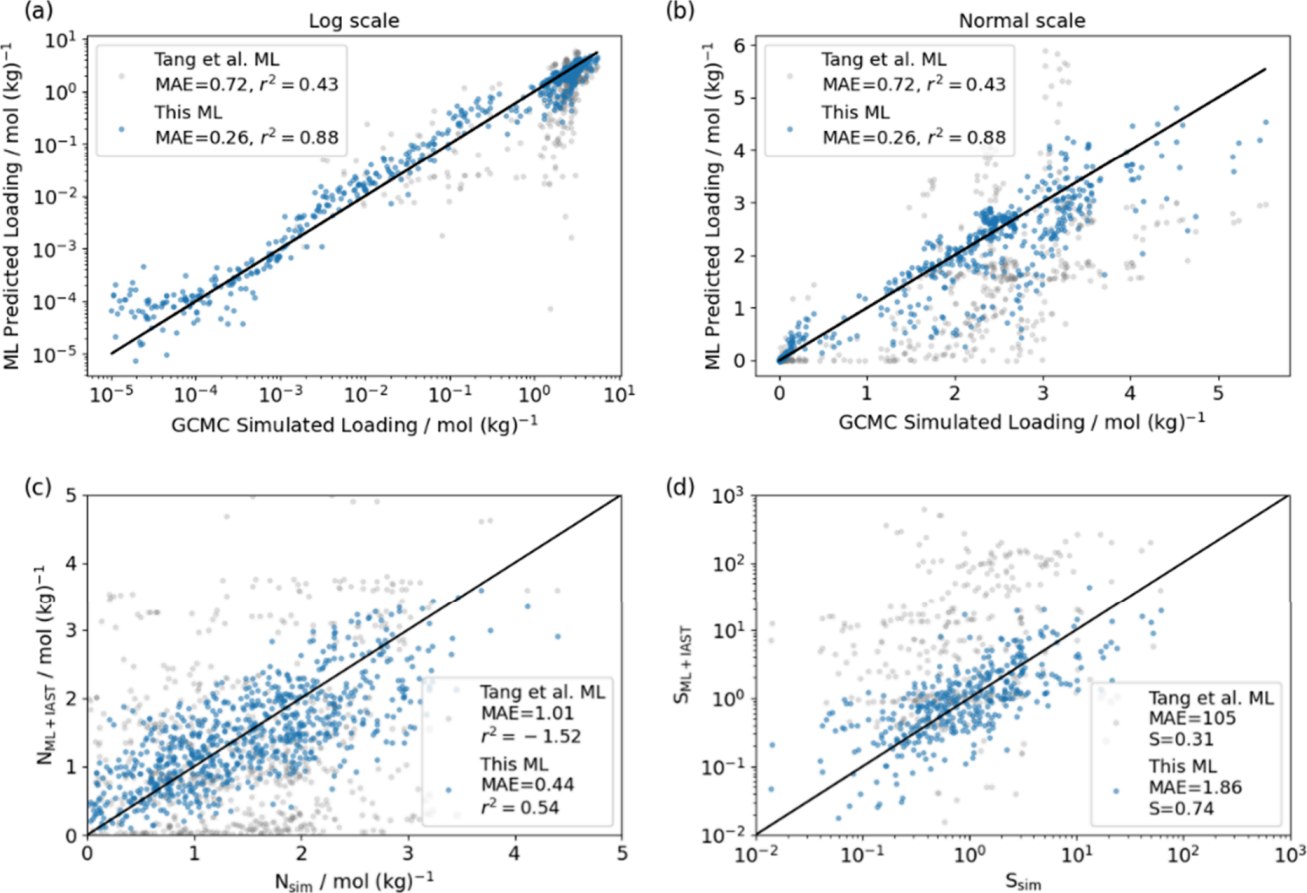
Single-component loading prediction of 12 near-azeotropic molecules in six MOFs at 300 K. Average loading parity plots of GCMC-simulated vs ML-predicted in (a) log scale and (b) normal scale. (c) Binary loadings and (d) selectivities of 66 binary mixtures in six MOFs simulated from direct binary GCMC simulation compared with calculated from ML-predicted single component isotherms with IAST. The MAE and Spearman’s coefficient (*S*) for the models in (c) and (d) are shown as insets.

We revisited this challenging test with our ensemble of ML models. Only two of the molecules from the 12 molecules simulated by Tang et al. were included in the training set for our ML model. As can be seen in Figure 9a,b, single-component isotherm loading predictions were significantly improved relative to those of earlier work, especially in the low-pressure regime. Our model had *r*^2^ = 0.88 and MAE = 0.26 mol/kg, which is encouraging for this set of unseen molecules. Using our improved ML-predicted single-component isotherms, we were able to make more accurate binary loading and selectivity predictions with IAST than the work of Tang et al. (Figure 9c,d, both with a much smaller MAE). Although predicting the precise numerical values for the adsorption selectivities remained difficult, we were able to gain valuable insights into the relative ranking of the MOFs, as evidenced by the Spearman’s coefficient of 0.74.

## 4 Discussions and Conclusions

In this paper, we have tackled the challenge of predicting equilibrium isotherms for arbitrary molecules in arbitrary MOFs, allowing the full adsorption space of these materials to be treated. It has recently been argued that a key factor limiting the impact of ML in chemistry is the availability of high-quality data.^54^ An important outcome of this paper is data from molecular simulations that greatly diversify the range of molecules for which isotherms in a large number of MOFs is available. This data not only allowed us to develop our own ML models but will hopefully catalyze similar model development by others.

Use of the ML models that we developed for arbitrary MOF–molecule pairs requires computing several descriptors and of the MOFs and molecules, including Henry’s constants for adsorption in the MOF. Obtaining Henry’s constants requires a molecular simulation, but this calculation is far less computationally demanding than simulating a complete adsorption isotherm. Efforts have been made to use ML to predict Henry’s constants for adsorption in MOFs without requiring detailed simulations,^23,55^ but it is not clear that these models are sufficiently accurate to be used reliably as features in models predicting complete isotherms.

We developed a classification model that predicts whether the adsorption of a molecule in an MOF can occur at physically significant levels. This model was mainly used to condition our data prior to training more quantitative models, but it could also be useful when considering MOFs for applications involving complex mixtures of many components.^56^ We also developed a model that predicts the complete single component isotherm at room temperature for arbitrary molecule–MOF pairs. Uncertainty in these estimates for both single component predictions and mixture predictions enabled by IAST was quantified using an ensemble of models rather than a single model.

It is important to clarify the limitations of our approach. Our training data was limited to molecules with molecular weights <100 g/mol that can be treated with the TraPPE FF. We have not tested our model for any molecules that cannot currently be described by this FF. Our GCMC simulations of adsorption assumed that each MOF was rigid and that molecule–MOF interactions could be defined with standard mixing rules. Our simulations also assumed that each MOF was free from defects. Examples are known from more detailed calculations where relaxing each of these assumptions makes a significant difference to adsorption properties. All real MOFs have at least so-called intrinsic flexibility due to atomic vibrations, and further flexibility effects can arise from adsorption-induced swelling or transitions.^38,57−59^ Molecular interactions with open metal sites can often not be accurately described with simple FFs.^60−63^ Cases are known in which the presence of local defects makes a decisive impact on adsorption properties.^64−66^ For each of these effects, more detailed simulations are possible to probe the quality of predictions for cases of particular interest. As an example, comparisons can be made between adsorption energies predicted by FFs and by DFT calculations.^37,60^ We view the aim of our current ML models as being to suggest specific candidates that can be examined in this more detailed way and not a tool that can be used in isolation. An interesting challenge for the future will be to augment training data at one level of approximation (such as the data set that we have introduced here) with judiciously chosen data from more detailed methods to improve the possibility of making accurate predictions about real materials.

Our data only consider adsorption at a single temperature. Many adsorption-based separations use temperature variations to cycle an adsorbent between different states. Yu et al. developed ML models predicting the temperature dependence of Henry’s constants for molecular adsorption in MOFs.^23^ These models could in principle be used in combination with the isotherm models that we have introduced here to predict temperature-dependent isotherms by assuming that the loading dependence of the heat of adsorption is negligible. We have not tested this concept.

Finally, our methods only provide information about equilibrium adsorption in MOFs. Interesting examples of effective separations of industrially relevant near-azeotropic molecular pairs in MOFs that rely on kinetic separations are known.^67^ Any analysis that considers only equilibrium isotherms cannot address the possible existence or absence of kinetic separations or diffusion limitations that would create challenges for equilibrium-based separations.

## Supporting Information Available

Details on the addition of state points to isotherms, spherical descriptor calculation, pyIAST modifications, parity plots for all regressors and binary mixtures, list of descriptors, and adsorbate information (PDF)
Numerical values of descriptors for MOF-molecule pairs, data of isotherms, and binary adsorption properties for near-azeotropic pairs (ZIP)
