# Supplementary material for: Efficient Exploration of Adsorption Space for Separations in Metal–Organic Frameworks Combining the Use of Molecular Simulations, Machine Learning, and Ideal Adsorbed Solution Theory
Source: J Phys Chem C Nanomater Interfaces. 2023 Sep 14;127(38):19229–39. doi: 10.1021/acs.jpcc.3c04533 (PMC10544990; doi:10.1021/acs.jpcc.3c04533)
Supplement: Supplementary file 1 — jp3c04533_si_001.pdf [file jp3c04533_si_001.pdf]

Supporting Information

Efficient Exploration of Adsorption Space for Separations in MOFs Combining the Use of Molecular Simulations, Machine Learning and IAST

Xiaohan Yu[1], Dai Tang[1], Jia Yuan Chng[1], David Sholl[1,2,*]

[1]*School of Chemical & Biomolecular Engineering, Georgia Institute of Technology, Atlanta, GA 30332, USA*
[2]*Oak Ridge National Laboratory, Oak Ridge, TN 37830, USA*

[*]Corresponding author. Email: shollds@ornl.gov

Table of Contents

S1.1 Addition of Intermediate State Points to Isotherms 2
S1.2 'Decreasing' and 'Large Uncertainty' Isotherm Examples 2
S1.3 Calculation of Diameter Descriptors 3
S1.4 pyIAST Modifications 4
S2 Supplementary Figures 5
S3 Supplementary Tables 19

In addition to the Supporting Information below, code that implements all models described in the manuscript is available via GitHub at https://github.com/tdytjd/mof_diverse_isotherm_prediction/. Three directories are under the GitHub repository. The 'classification' directory and the 'regression' directory correspond to Section 3. I and 3. II respectively. The 'IAST' directory has the scripts of modified pyIAST code and calculation of binary adsorption properties. Ten supplementary data files are also available in a ZIP file accompanying this publication. The names of the data files and what they represent in this ZIP file are listed below.

- Data S1.csv: Data used for the classification of Set 1
- Data S2.csv: Data used for the classification of Set 2
- Data S3.csv: Data used for the regression of Set 1
- Data S4.csv: Data used for the regression Set 2
- Data S5.csv: Data used for the regression of extra 12 molecules in 6 MOFs
- Data S6.json: Full Isotherm Database
- Data S7.xlsx: Binary adsorption properties calculated for the 13 near-azeotropic pairs
- Data S8.csv: Binary adsorption properties calculated for the extra 12 molecules in 6 MOFs
- Data S9.xlsx: Tabulated data of all figures in the manuscript
- Data S10: Example Simulation Input Files

S1.1 Addition of Intermediate State Points to Isotherms

To obtain more reliable fitting of single-component isotherms, we added intermediate state points to some isotherms. To determine when this step is needed, each state point is assumed to follow a Gaussian distribution, where the mean and standard deviation of the distribution are the loading mean and standard deviation of the RASPA simulation. For each state point, we sampled 10 values by drawing from the distribution 10 times. Each set of sampled loadings was fitted to continuous isotherm functions as described in the main manuscript, and we calculated the selectivities of a near-azeotropic molecule pair from 100 possible combinations of the two sets of 10 isotherms. When the uncertainty of the selectivities is larger than 25%, we calculated the pressure corresponding to 0.35*saturation loading for each isotherm. We then simulated a new state point at the average of these 10 calculated pressures. We double checked the calculated pressures visually and a small number of calculated pressures were updated to more reasonable values manually. We completed this process for all 13 near-azeotropic pairs in 335 MOFs.

S1.2 'Decreasing' and 'Large Uncertainty' Isotherm Examples

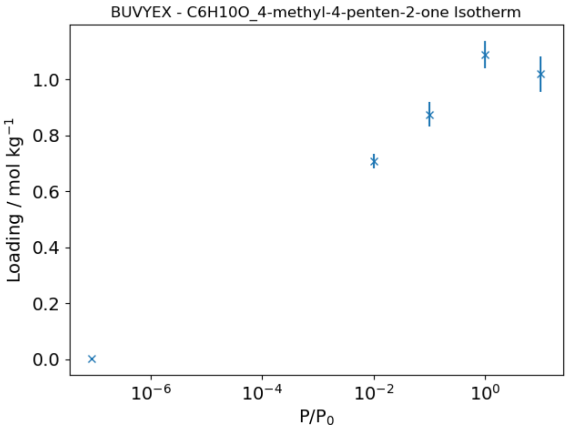

Fig. S1: GCMC data for methyl-4-penten-2-one in BUVYEX isotherm marked as 'Decreasing'

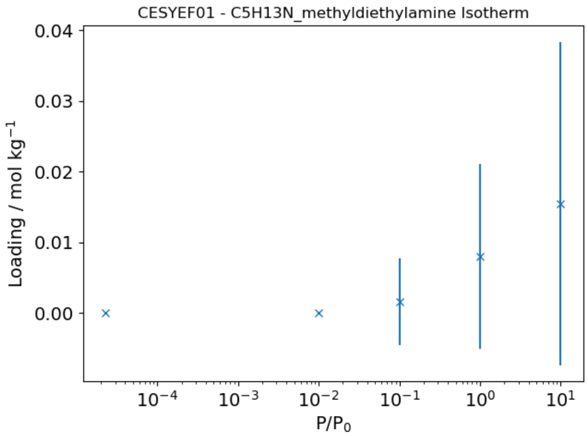

Fig. S2: GCMC data for methyldiethylamine in CESYEF01 isotherm marked as 'Large Uncertainty'

S1.3 Calculation of Diameter Descriptors

We used three spherical descriptors for each adsorbates in the ML training calculated using the minimum enclosed ellipsoid method[1]. This method involves finding the smallest ellipsoid that fully encloses the convex hull formed by the 3D coordinates of the atom centers of an adsorbate molecule. The 3D coordinates of cyclic molecules are obtained after DFT optimization at the PBE-D3 level. The 3D coordinates of non-cyclic molecules that are neither linear nor spherical are obtained from their most elongated conformations. In these conformations, all bond angles along the molecule's backbone are set to their force field's equilibrium angles. The enclosing ellipsoid has three principal axes, and the three diameter descriptors are the lengths of these axes. Code to perform this calculation can be found at https://github.com/tdytjd/mof_diverse_isotherm_prediction/.

S1.4 pyIAST Modifications

We used pyIAST to fit isotherms, and we modified the fitting process to give more accurate results in the low-pressure regime. To make quantitative predictions about adsorption selectivity with IAST, the accuracy of isotherm fitting in low-pressure regime is essential[2]. PyIAST calculated the root mean square error (RMSE, equation (1)) between the fitted loadings and the actual loadings, and we chose the adsorption model with the lowest RMSE.

$$RMSE = \sqrt[2]{\frac{\sum_i (Fitted\ Loading_i - Acutal\ Loading_i)^2}{n}} \quad (1)$$

When calculating the RMSE, the impact of lower-loading errors on the value is diminished due to their significantly smaller magnitudes. To account for this effect, we used the $\log_{10}$(loading) values for loadings < 0.1 mol/kg when calculating the RSME in our modified algorithm to make their magnitudes comparable to other state points and emphasize the accuracy of fitting in the low-pressure regime. Fig. S3 shows that the modified fitting significantly reduces the relative errors in the low-pressure regime while the distribution of the relative errors of other points is similar to the distribution using the original pyIAST fitting. In Fig. S4, the selectivities were calculated from two sets of fitted isotherms, which were pyIAST fitting and modified fitting. The loadings in the low-pressure regime were fitted accurately using the modified algorithm, which led to a very different selectivity. Modified code can be found at https://github.com/tdytjd/mof_diverse_isotherm_prediction/IAST.

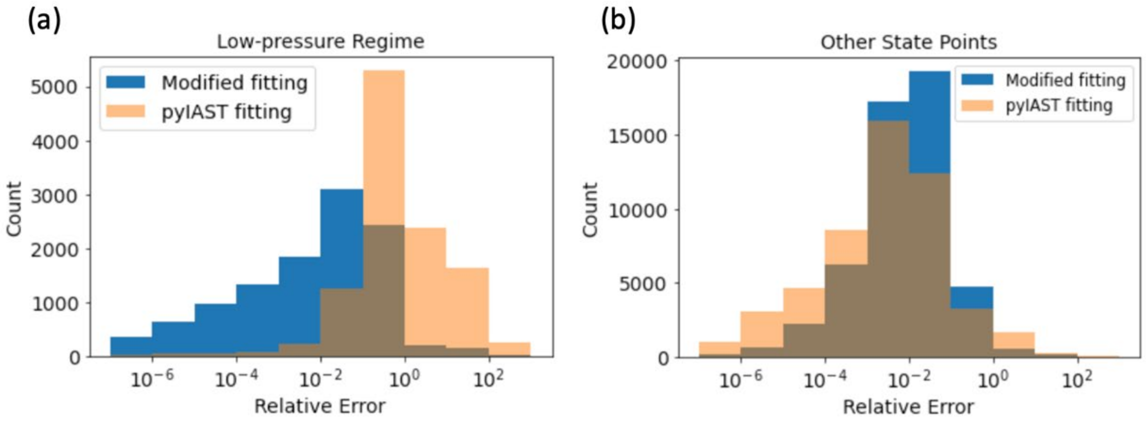

Fig. S3: Comparison of relative errors of all fitting results in (a) low-pressure regime and (b) other state points

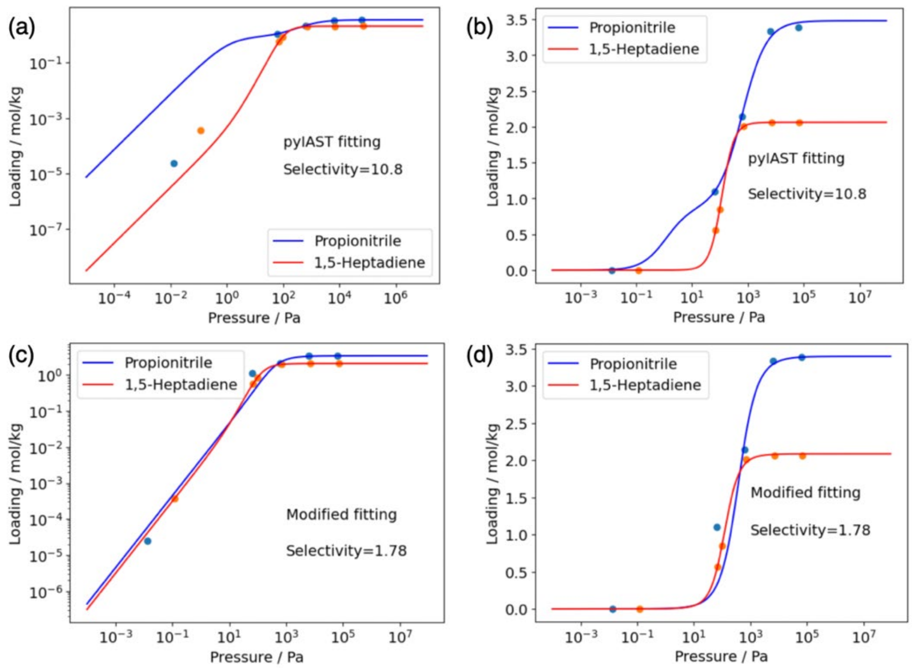

Fig. S4: Comparison of isotherm fittings of propionitrile and 1,5-heptadien in MOF ACOLIP using pyIAST (a,b) and modified algorithm (c,d). The isotherms are shown in log scale (a,c) and normal scale (b,d).

S2 Supplementary Figures

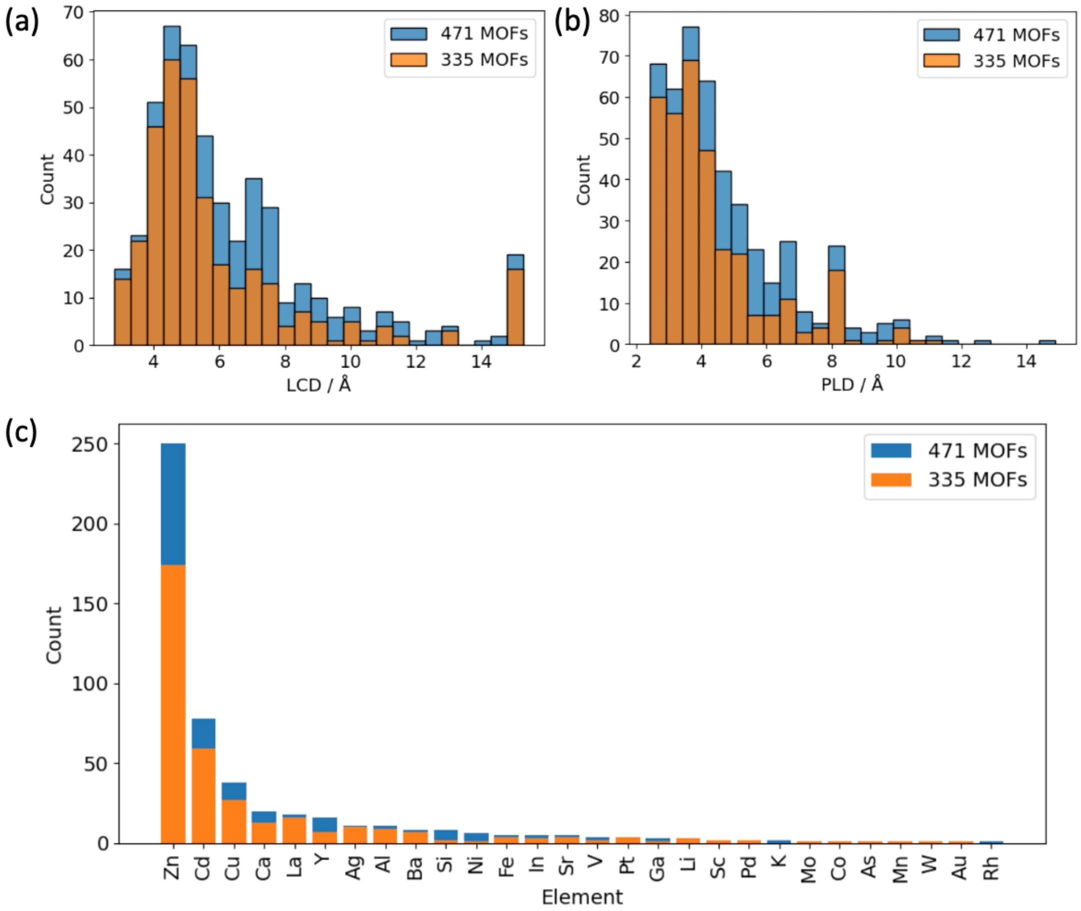

Fig. S5: Distributions of (a) LCD, (b) PLD and (c) metal centers before and after the number of MOFs considered.

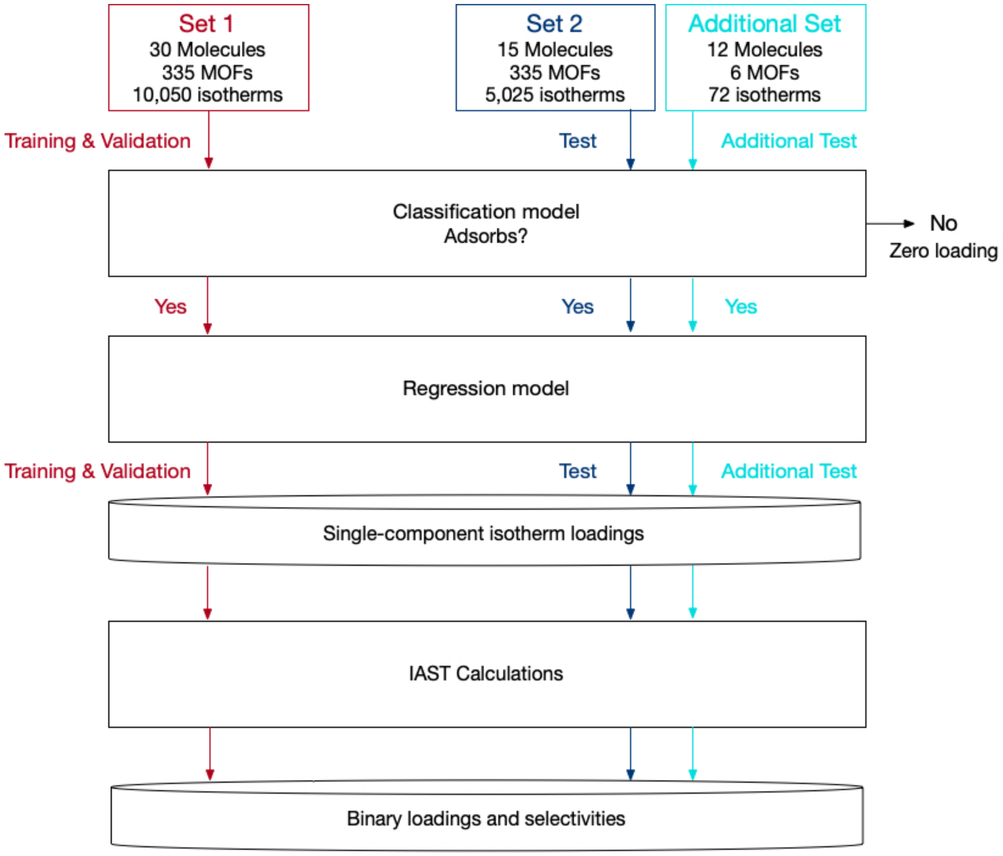

Fig. S6: The workflow of our ML approach for predicting binary adsorption for the MOF/molecule pairs

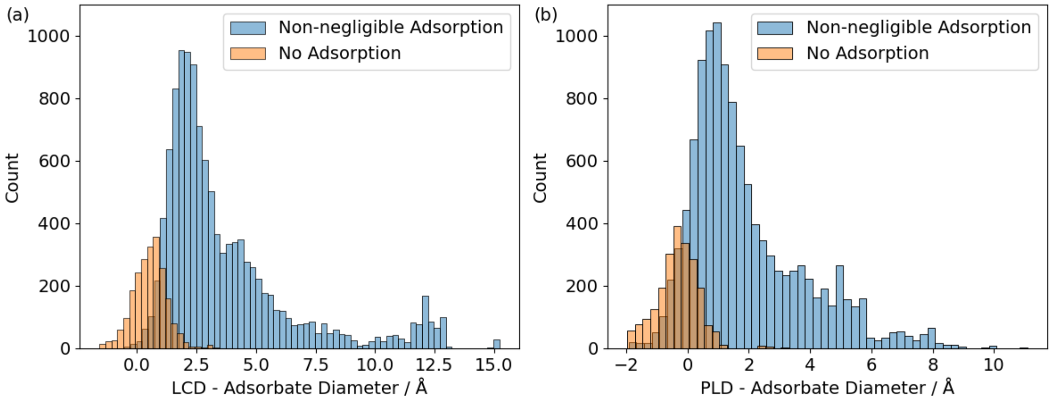

Fig. S7: The distributions of the difference between the pore size of the MOFs ((a) LCD, (b) PLD) and the smallest diameter of the adsorbate. The histogram of MOF/adsorbate pairs with non-negligible adsorption is shown in blue. The histogram of MOF/adsorbate pairs with no adsorption detected is shown in orange.

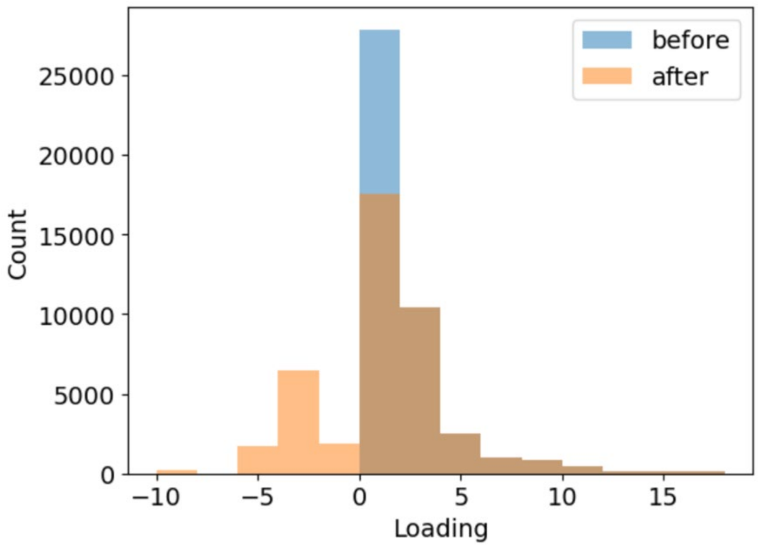

Fig. S8: Distribution of the target values (loadings) for ML predictions before and after scaling the original loadings obtained from GCMC simulations

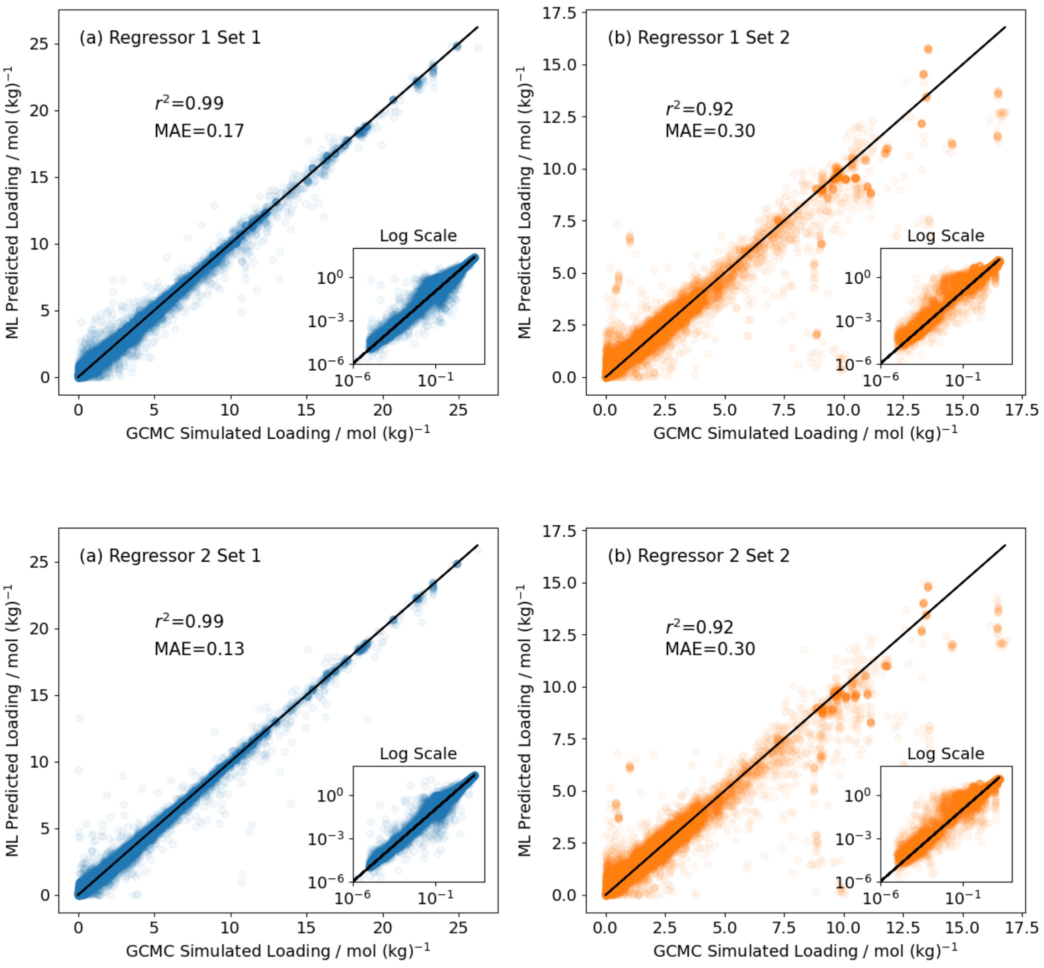

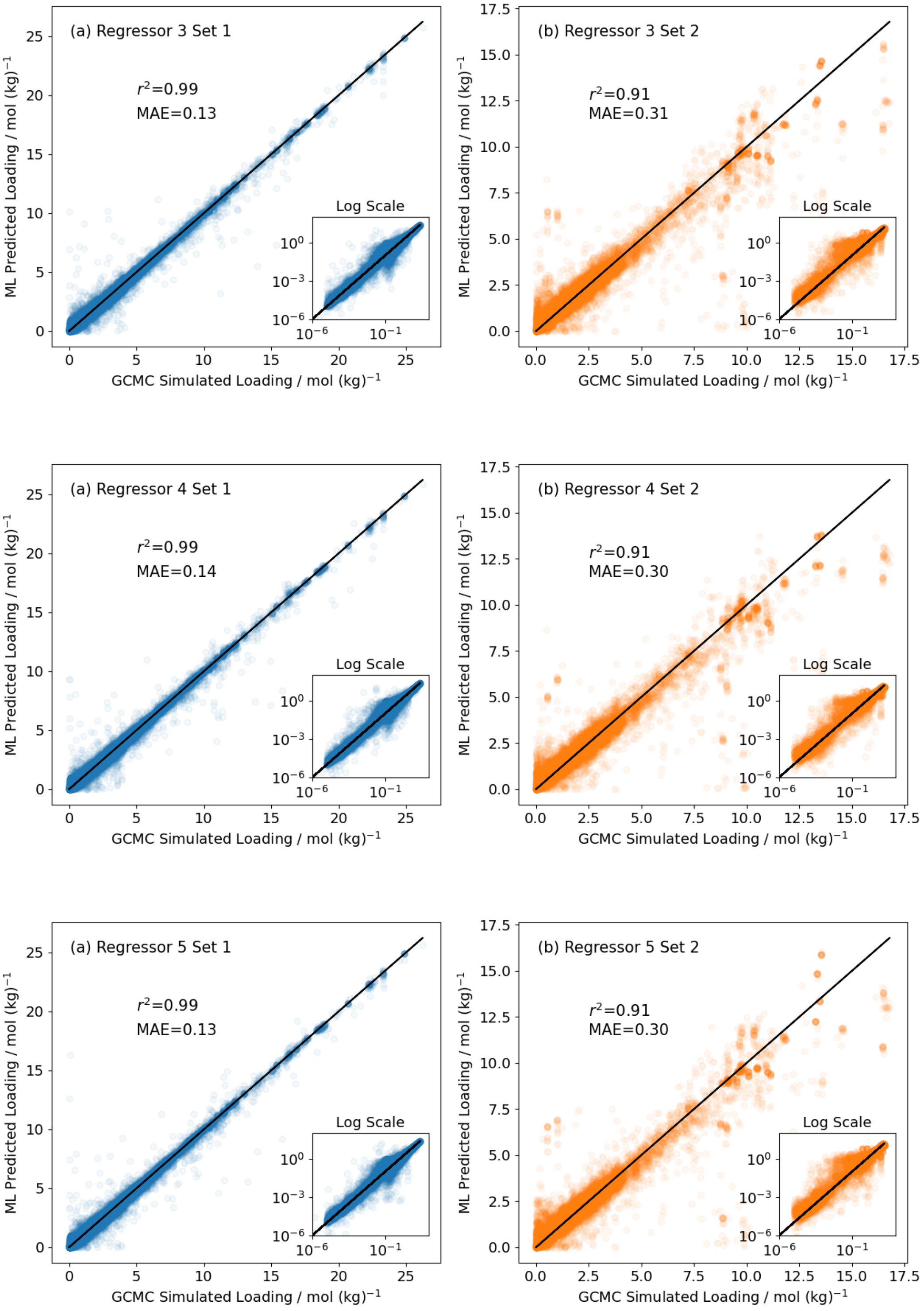
(a) Regressor 3 Set 1
r^2=0.99
MAE=0.13
(b) Regressor 3 Set 2
r^2=0.91
MAE=0.31
(a) Regressor 4 Set 1
r^2=0.99
MAE=0.14
(b) Regressor 4 Set 2
r^2=0.91
MAE=0.30
(a) Regressor 5 Set 1
r^2=0.99
MAE=0.13
(b) Regressor 5 Set 2
r^2=0.91
MAE=0.30
Log Scale
ML Predicted Loading / mol (kg)$^{-1}$
GCMC Simulated Loading / mol (kg)$^{-1}$

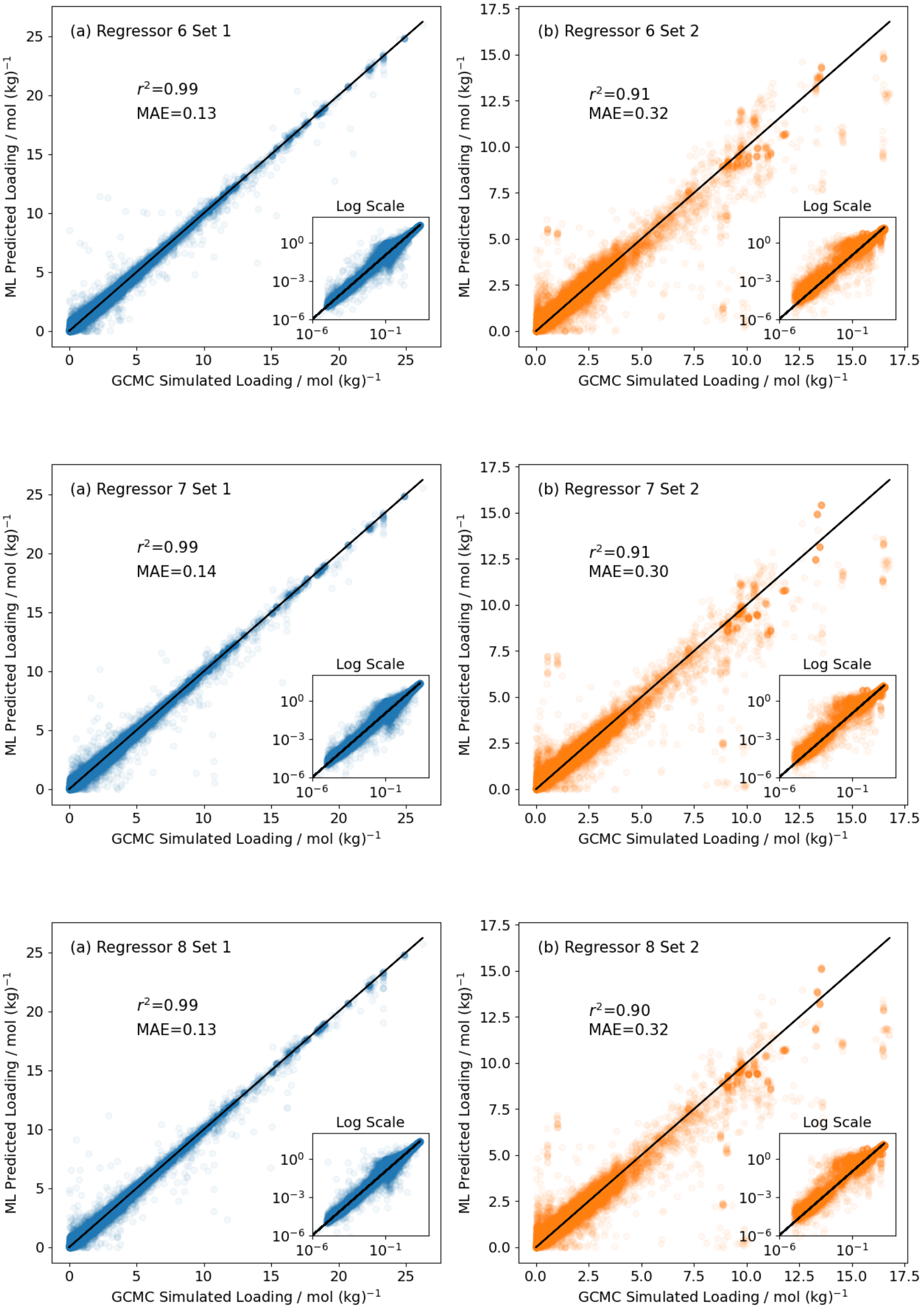
(a) Regressor 6 Set 1
r^2=0.99
MAE=0.13
(b) Regressor 6 Set 2
r^2=0.91
MAE=0.32
(a) Regressor 7 Set 1
r^2=0.99
MAE=0.14
(b) Regressor 7 Set 2
r^2=0.91
MAE=0.30
(a) Regressor 8 Set 1
r^2=0.99
MAE=0.13
(b) Regressor 8 Set 2
r^2=0.90
MAE=0.32
Log Scale
ML Predicted Loading / mol (kg)$^{-1}$
GCMC Simulated Loading / mol (kg)$^{-1}$

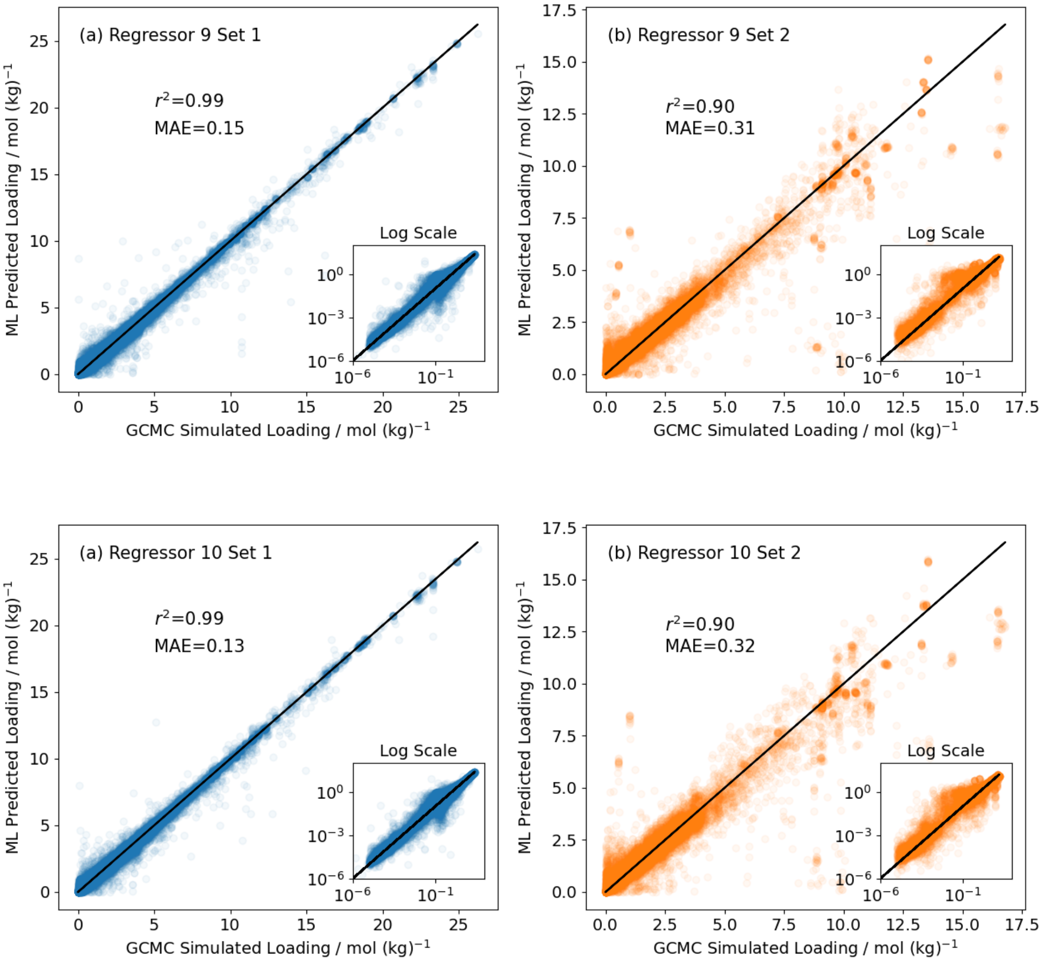

Fig. S9: Parity plots of 10 individual regressors in the ensemble. ML predicted loadings compared with GCMC simulated loadings of 30 molecules in (a) Set 1 and 15 molecules in (b) Set 2 in 335 MOFs at 300K.

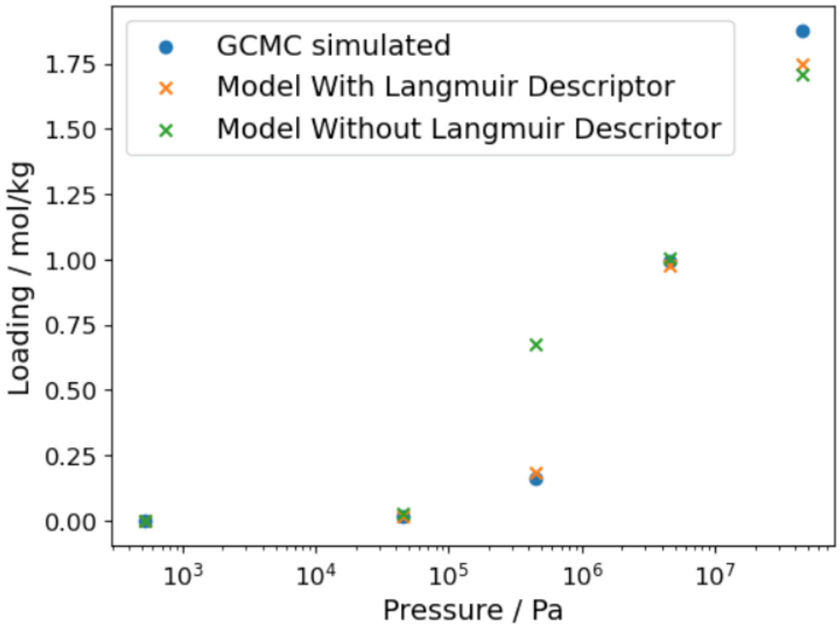

Fig. S10: PELNEA01/methane adsorption prediction by models trained with and without Langmuir descriptor.

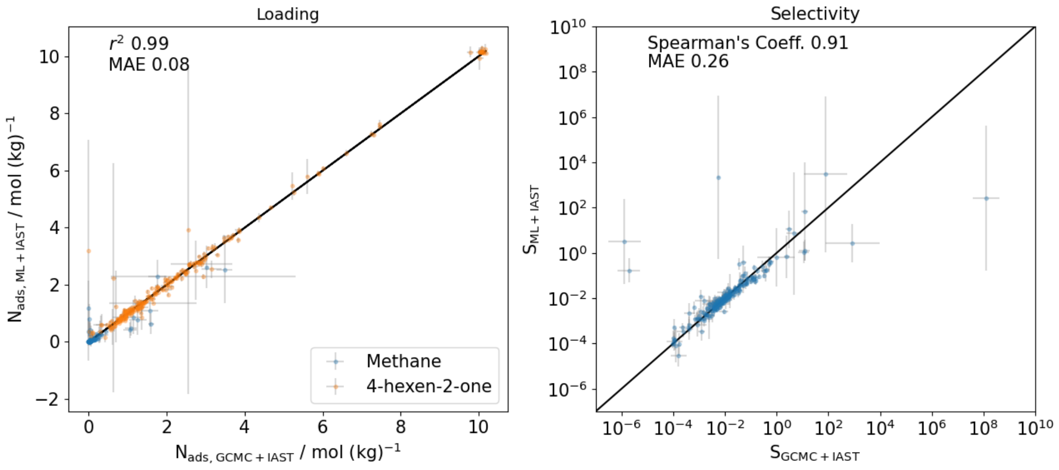

Fig. S11: Parity plots of loadings (left) and selectivities (right) of equimolar methane and 4-hexen-2-one separation in 335 MOFs

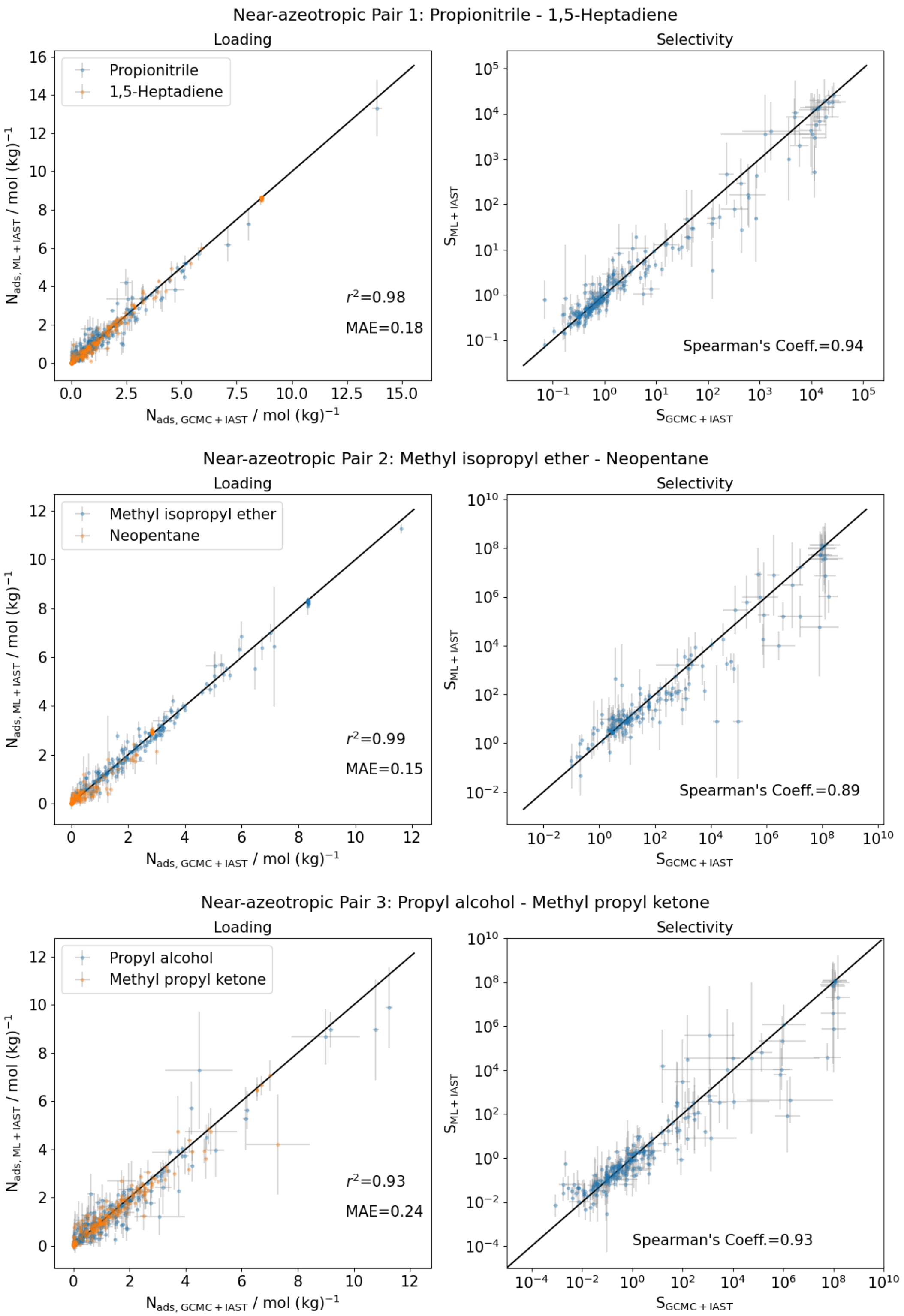
Near-azeotropic Pair 1: Propionitrile - 1,5-Heptadiene
Loading
Selectivity
Propionitrile
1,5-Heptadiene
r^2=0.98
MAE=0.18
Spearman's Coeff.=0.94
$N_{ads, ML+IAST}$ / mol (kg)$^{-1}$
$N_{ads, GCMC+IAST}$ / mol (kg)$^{-1}$
$S_{ML+IAST}$
$S_{GCMC+IAST}$
Near-azeotropic Pair 2: Methyl isopropyl ether - Neopentane
Loading
Selectivity
Methyl isopropyl ether
Neopentane
r^2=0.99
MAE=0.15
Spearman's Coeff.=0.89
$N_{ads, ML+IAST}$ / mol (kg)$^{-1}$
$N_{ads, GCMC+IAST}$ / mol (kg)$^{-1}$
$S_{ML+IAST}$
$S_{GCMC+IAST}$
Near-azeotropic Pair 3: Propyl alcohol - Methyl propyl ketone
Loading
Selectivity
Propyl alcohol
Methyl propyl ketone
r^2=0.93
MAE=0.24
Spearman's Coeff.=0.93
$N_{ads, ML+IAST}$ / mol (kg)$^{-1}$
$N_{ads, GCMC+IAST}$ / mol (kg)$^{-1}$
$S_{ML+IAST}$
$S_{GCMC+IAST}$

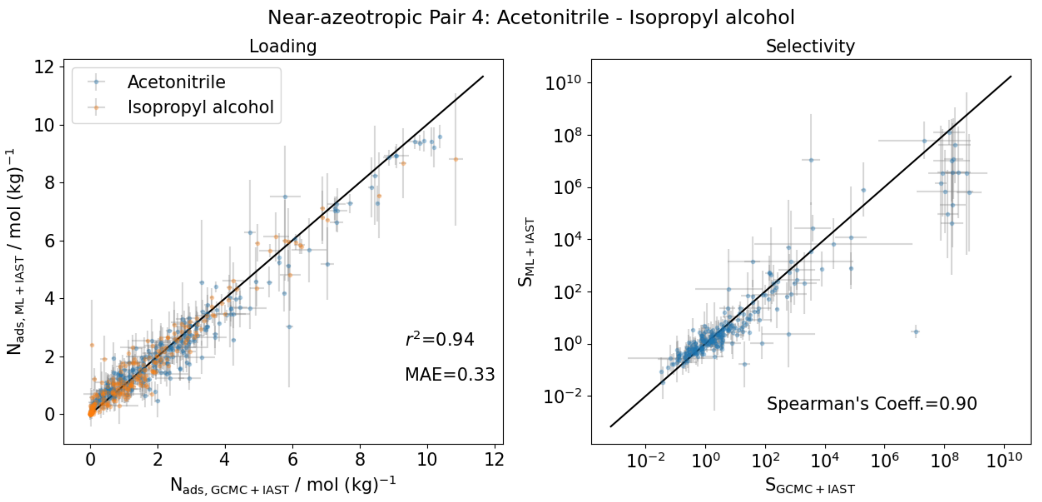
Near-azeotropic Pair 4: Acetonitrile - Isopropyl alcohol
Loading
Selectivity
Acetonitrile
Isopropyl alcohol
r^2=0.94
MAE=0.33
Spearman's Coeff.=0.90
$N_{ads, ML+IAST}$ / mol (kg)$^{-1}$
$N_{ads, GCMC+IAST}$ / mol (kg)$^{-1}$
$S_{ML+IAST}$
$S_{GCMC+IAST}$

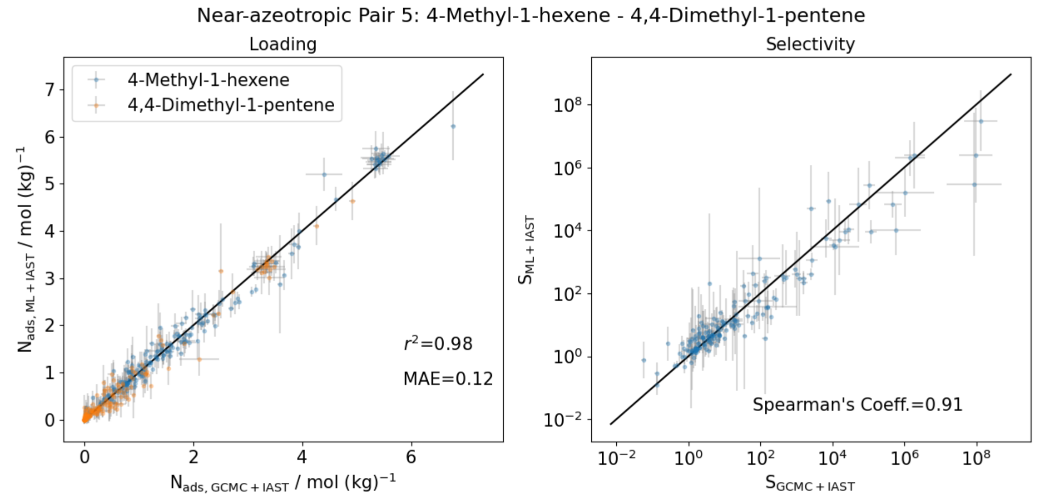
Near-azeotropic Pair 5: 4-Methyl-1-hexene - 4,4-Dimethyl-1-pentene
Loading
Selectivity
4-Methyl-1-hexene
4,4-Dimethyl-1-pentene
r^2=0.98
MAE=0.12
Spearman's Coeff.=0.91
$N_{ads, ML+IAST}$ / mol (kg)$^{-1}$
$N_{ads, GCMC+IAST}$ / mol (kg)$^{-1}$
$S_{ML+IAST}$
$S_{GCMC+IAST}$

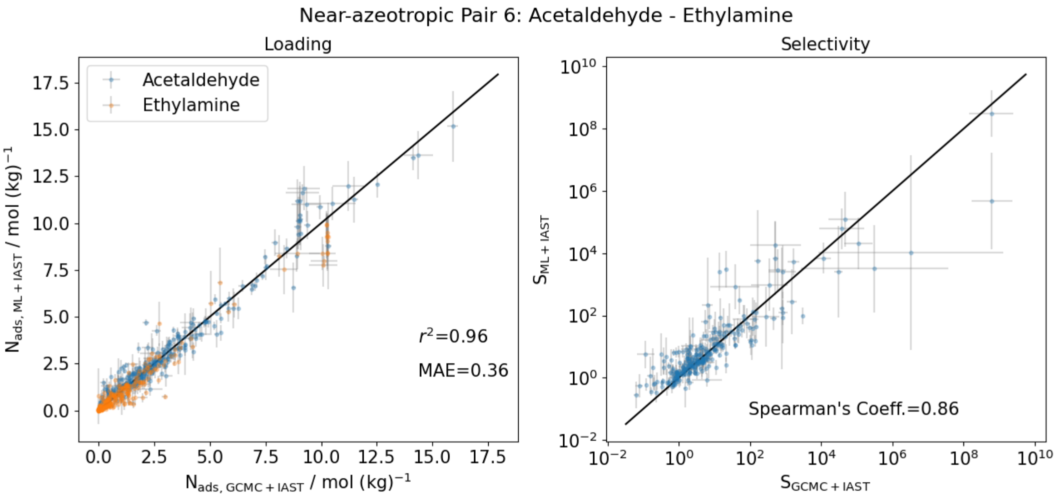
Near-azeotropic Pair 6: Acetaldehyde - Ethylamine
Loading
Selectivity
Acetaldehyde
Ethylamine
r^2=0.96
MAE=0.36
Spearman's Coeff.=0.86
$N_{ads, ML+IAST}$ / mol (kg)$^{-1}$
$N_{ads, GCMC+IAST}$ / mol (kg)$^{-1}$
$S_{ML+IAST}$
$S_{GCMC+IAST}$

Near-azeotropic Pair 7: Acetaldehyde - Dimethylamine

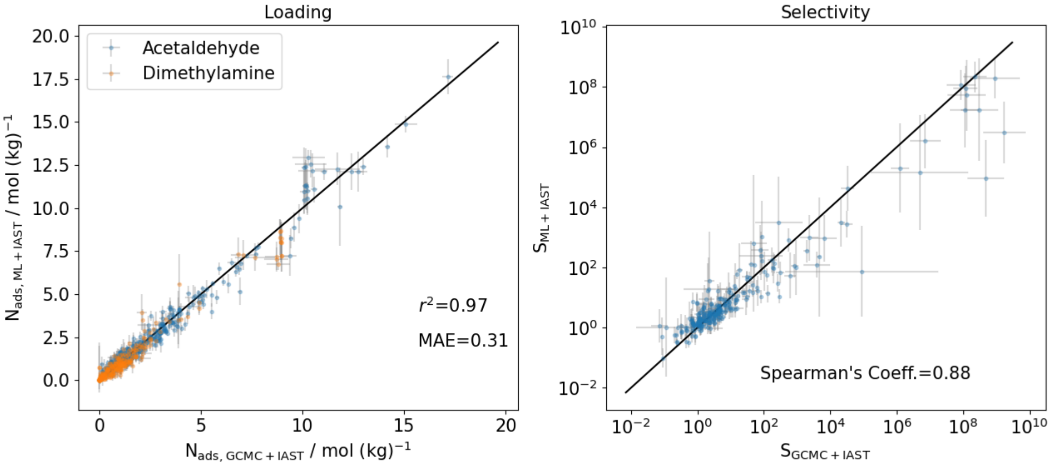

Near-azeotropic Pair 8: Methyl propyl ether - 2-Pentene

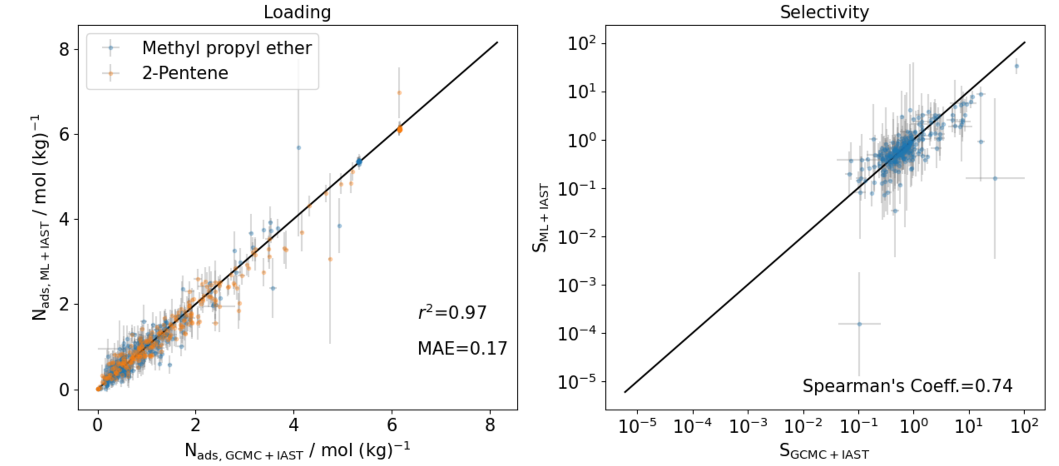

Near-azeotropic Pair 9: Propene - Propane

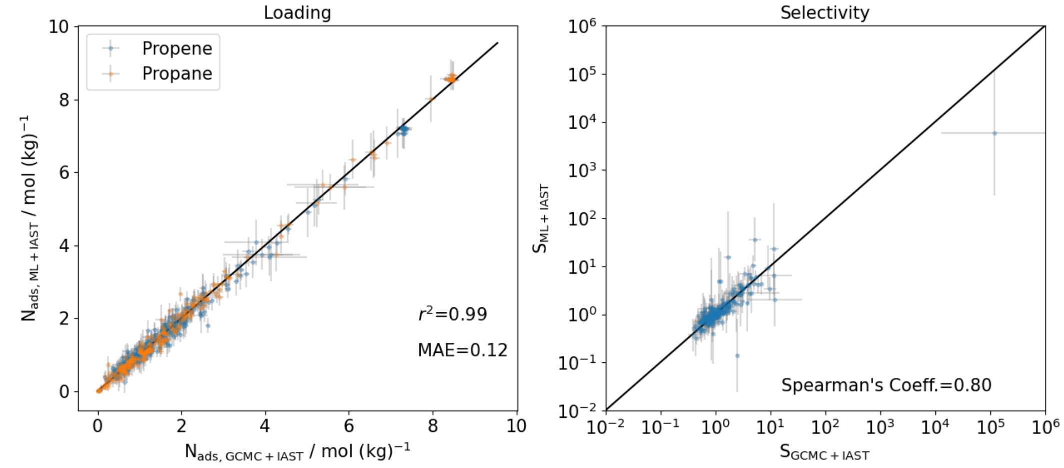

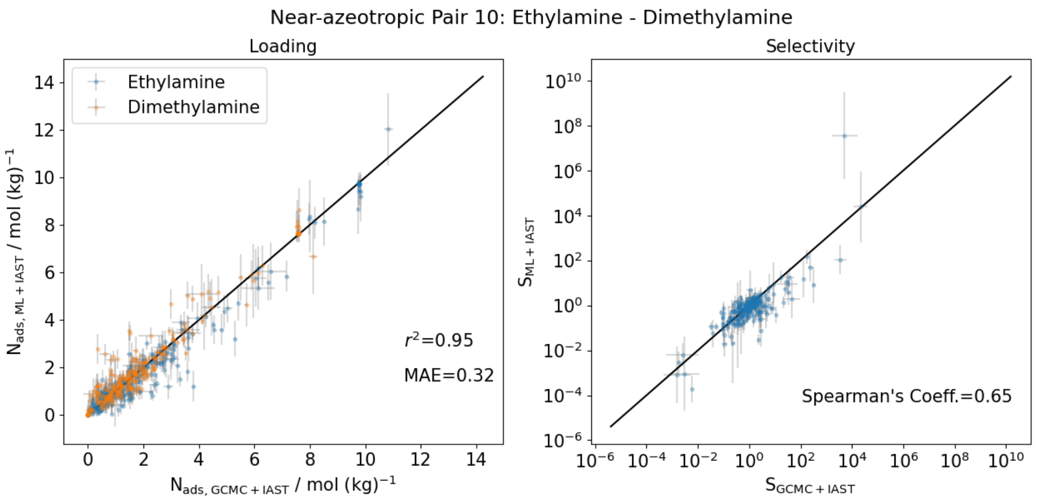
Near-azeotropic Pair 10: Ethylamine - Dimethylamine
Loading
Selectivity
Ethylamine
Dimethylamine
r^2=0.95
MAE=0.32
Spearman's Coeff.=0.65
$N_{ads, ML+IAST}$ / mol (kg)$^{-1}$
$N_{ads, GCMC+IAST}$ / mol (kg)$^{-1}$
$S_{ML+IAST}$
$S_{GCMC+IAST}$

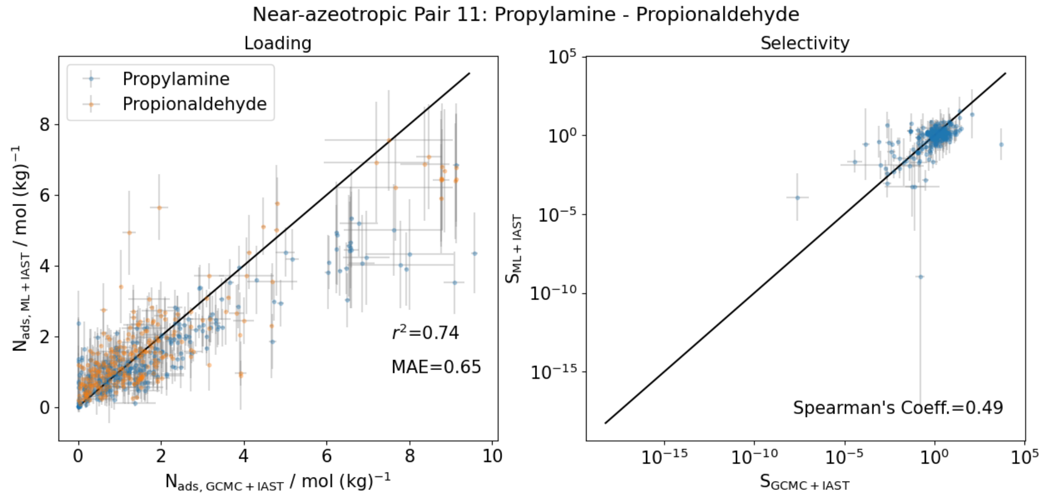
Near-azeotropic Pair 11: Propylamine - Propionaldehyde
Loading
Selectivity
Propylamine
Propionaldehyde
r^2=0.74
MAE=0.65
Spearman's Coeff.=0.49
$N_{ads, ML+IAST}$ / mol (kg)$^{-1}$
$N_{ads, GCMC+IAST}$ / mol (kg)$^{-1}$
$S_{ML+IAST}$
$S_{GCMC+IAST}$

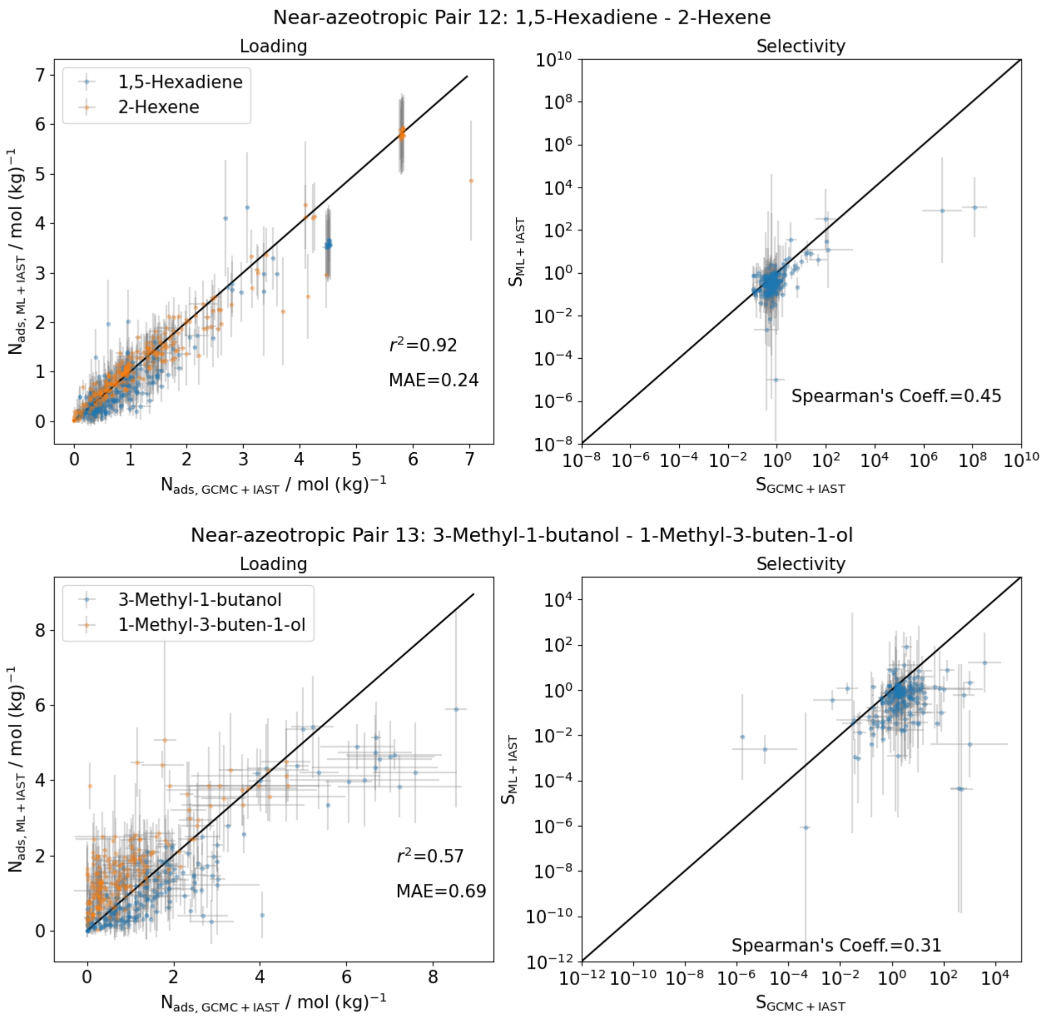

Fig. S12: Parity plots of binary loadings and selectivities with uncertainties of each near-azeotropic pair

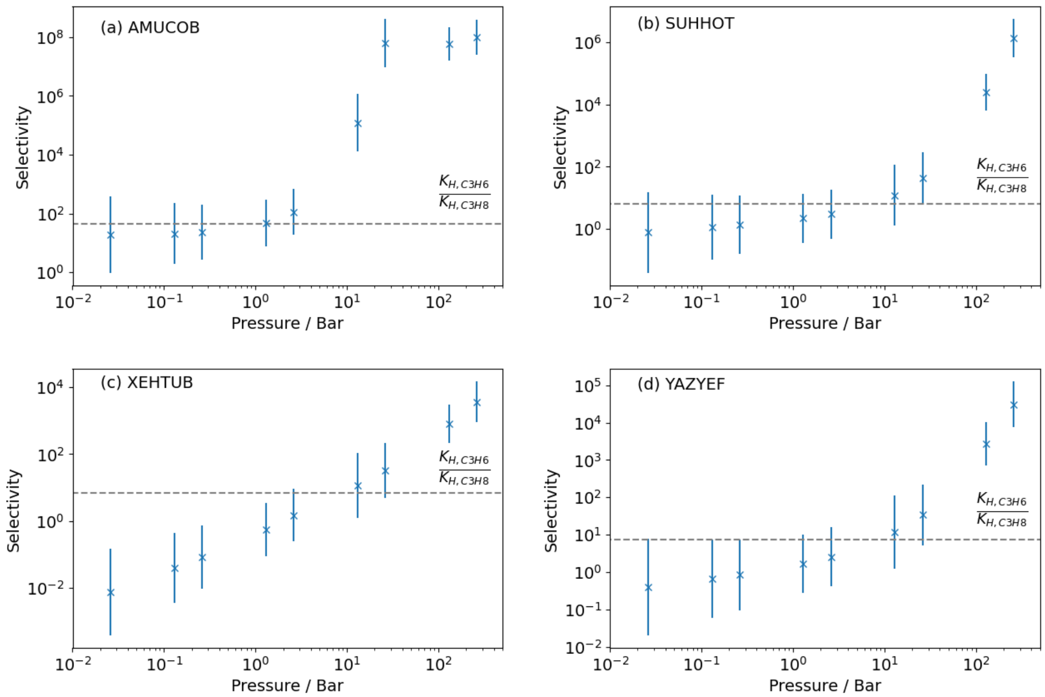

Fig. S13: Selectivities of propene/propane separation from equimolar bulk mixtures at 300 K as a function of pressure in the four best performing MOFs as defined by the selectivity metric in Figure 6: (a) AMUCOB, (b) SUHHOT, (c) XEHTUB, (d) YAZYEF. All data shown is from applying IAST to ML-predicted single component isotherms.

S3 Supplementary Tables

Table S1 Information of the 45 molecules simulated

Set 1

Name	Formula	MW / g/mol	Tc / K	Pc / bar	omega	Tb / K	FF
methane	CH4	16.04	352.93	55.90	0.13	111.60	UA
hydrogen cyanide	CHN	27.03	447.25	53.05	0.33	272.38	EH
ethene	C2H4	28.05	298.46	49.66	0.12	169.50	UA
ethane	C2H6	30.07	381.79	50.32	0.22	183.99	UA
acetonitrile	C2H3N	41.05	537.42	47.22	0.36	347.04	EH
propene	C3H6	42.08	393.00	45.73	0.16	225.60	UA
acetaldehyde	C2H4O	44.05	470.13	53.98	0.29	298.84	UA
propane	C3H8	44.10	416.48	44.60	0.24	230.06	UA
dimethylamine	C2H7N	45.08	449.30	53.96	0.29	297.91	EH
ethylamine	C2H7N	45.08	456.14	52.30	0.28	298.48	EH
dimethyl ether	C2H6O	46.07	410.11	48.44	0.23	250.75	UA
propionitrile	C3H5N	55.08	558.86	42.25	0.36	379.64	EH
acetone	C3H6O	58.08	504.90	46.14	0.30	316.93	UA
isobutane	C4H10	58.12	443.01	38.86	0.20	271.54	UA
isopropyl alcohol	C3H8O	60.10	515.58	48.73	0.58	350.66	UA
propyl alcohol	C3H8O	60.10	548.73	52.15	0.53	365.92	UA
2-pentene	C5H10	70.13	485.00	36.50	0.25	310.18	UA
isopentane	C5H12	72.15	474.76	34.74	0.24	303.10	UA
neopentane	C5H12	72.15	470.54	35.45	0.20	308.71	UA
methyl isopropyl ether	C4H10O	74.12	464.96	38.54	0.25	307.80	UA
methyl propyl ether	C4H10O	74.12	478.87	38.38	0.29	309.54	UA
methyl propyl ketone	C5H10O	86.13	564.47	36.93	0.35	368.68	UA
2,3-dimethylbutane	C6H14	86.18	503.07	31.56	0.24	339.52	UA
3-methylpentane	C6H14	86.18	504.84	31.14	0.28	334.33	UA
methyl tert-butyl ether	C5H12O	88.15	512.69	34.05	0.27	325.83	UA
1,5-heptadiene	C7H12	96.17	536.77	30.48	0.30	376.59	UA
4-hexen-2-one	C6H10O	98.14	559.66	36.98	0.45	409.87	UA
4-methyl-4-penten-2-one	C6H10O	98.14	583.96	32.18	0.43	399.99	UA
4,4-dimethyl-1-pentene	C7H14	98.19	524.36	29.55	0.25	353.26	UA
4-methyl-1-hexene	C7H14	98.19	528.16	29.39	0.30	355.50	UA

Set 2

Name	Formula	MW / g/mol	Tc / K	Pc / bar	omega	Tb / K	FF
1-butene	C4H8	56.11	430.18	40.68	0.2	274.52	UA
isobutene	C4H8	56.11	433.53	40.52	0.2	272.11	UA
propionaldehyde	C3H6O	58.08	505.8	49.25	0.3	325.54	UA
propylamine	C3H9N	59.11	489.14	48.09	0.31	321.14	EH
dimethylethylamine	C4H11N	73.14	478.41	36.65	0.26	306.63	EH
1,5-hexadiene	C6H10	82.15	496.8	33.99	0.23	342.02	UA

2-hexene	C6H12	84.16	514.29	32.56	0.29	342.81	UA
1-methyl-3-buten-1-ol	C5H10O	86.13	547.15	41.11	0.72	401.81	UA
2,2-dimethylbutane	C6H14	86.18	502.04	32	0.24	326.25	UA
2-methylpentane	C6H14	86.18	504.84	31.14	0.28	336	UA
methyldiethylamine	C5H13N	87.16	502.51	33.18	0.31	334.84	EH
3-methyl-1-butanol	C5H12O	88.15	560.27	38.47	0.66	400.78	UA
1,3,5-heptatriene	C7H10	94.16	572.52	30.09	0.29	378.21	UA
4-methyl-4-pentenal	C6H10O	98.14	580.07	32.39	0.39	401.87	UA
5-hexen-2-one	C6H10O	98.14	598.2	35.16	0.46	405.39	UA

Table S2 Information of the 13 near-azeotropic pairs considered

Set	Pair no.	Component A	T_b / K	Component B	T_b / K	$\triangle T_b$ / K
Set 1	1	propionitrile	379.64	1,5-heptadiene	376.59	3.05
	2	methyl isopropyl ether	307.80	neopentane	308.71	0.91
	3	propyl alcohol	365.92	methyl propyl ketone	368.68	2.76
	4	acetonitrile	347.04	isopropyl alcohol	350.66	3.62
	5	4-methyl-1-hexene	355.50	4,4-dimethyl-1-pentene	353.26	2.24
	6	acetaldehyde	298.84	ethylamine	298.48	0.36
	7	acetaldehyde	298.84	dimethylamine	297.91	0.92
	8	methyl propyl ether	309.54	2-pentene	310.18	0.64
	9	propene	225.60	propane	230.06	4.46
	10	ethylamine	298.48	dimethylamine	297.91	0.56
Set 2	11	propylamine	321.14	propionaldehyde	325.54	4.41
	12	1,5-hexadiene	342.02	2-hexene	342.81	0.79
	13	3-methyl-1-butanol	400.78	1-methyl-3-buten-1-ol	401.81	1.03

Table S3 Descriptor information

Descriptor Name	Number of Descriptors
Geometrical descriptors (PLD, LCD, VF)	3
Potential Energy Surface (PES) descriptors	28
logK	1
Pressure	1
Molecular critical properties (Tc, Pc, omega)	3
xlogP	1
Spherical descriptors (diameters)	3
Langmuir descriptor	1
Total	41

Table S4 List of hyperparameters considered during ML training

hyperparameters	values
n_estimators	200, 300, 500
max_depth	4,5,6
learning_rate	0.01, 0.1, 0.15, 0.2

Table S5 Importance of the 41 descriptors. The importance type is 'gain', indicating the improvement in accuracy brought by a descriptor to the branches it is on.

Descriptor	Importance	Uncertainty
-700.0 ~ -600.0	0.388	0.026
0.0 ~ inf	0.208	0.026
Langmuir descriptor	0.112	0.006
VF	0.052	0.005
diameter_1	0.025	0.005
-600.0 ~ -500.0	0.023	0.008
xlogP	0.021	0.003
pressure	0.016	0.001
-inf ~ -2600.0	0.015	0.003
Tc	0.014	0.001
diameter_2	0.012	0.001
-1300.0 ~ -1200.0	0.011	0.003

Pc	0.011	0.002
-1600.0 ~ -1500.0	0.007	0.002
logK	0.007	0.001
-1200.0 ~ -1100.0	0.006	0.001
-2300.0 ~ -2200.0	0.006	0.001
diameter_3	0.005	0.001
-2200.0 ~ -2100.0	0.005	0.001
-400.0 ~ -300.0	0.005	0.002
w	0.004	0.001
-2100.0 ~ -2000.0	0.004	0.001
-1100.0 ~ -1000.0	0.003	0.002
-1400.0 ~ -1300.0	0.003	0.001
LCD	0.003	0.000
-500.0 ~ -400.0	0.003	0.001
-1000.0 ~ -900.0	0.003	0.001
-1500.0 ~ -1400.0	0.003	0.001
-800.0 ~ -700.0	0.003	0.000
-300.0 ~ -200.0	0.002	0.000
-900.0 ~ -800.0	0.002	0.000
-200.0 ~ -100.0	0.002	0.001
-2600.0 ~ -2500.0	0.002	0.000
-1700.0 ~ -1600.0	0.002	0.000
-100.0 ~ 0.0	0.002	0.000
-1800.0 ~ -1700.0	0.002	0.000
-2400.0 ~ -2300.0	0.002	0.000
-1900.0 ~ -1800.0	0.002	0.000
-2000.0 ~ -1900.0	0.002	0.000
PLD	0.002	0.000
-2500.0 ~ -2400.0	0.001	0.000

Table S6 15 top performing MOFs for propene/propane separation identified by IAST calculations using GCMC-simulated and ML-predicted single-component isotherms, ranked by adsorption selectivity. Materials that appear on both lists are indicated in italics.

Rank	GCMC	ML
1	*AMUCOB*	*AMUCOB*
2	SUHHOT	*BERFIP*
3	*XEHTUB*	*XEHTUB*
4	*YAZYEF*	MOGVAG
5	ICANEH	*XOVVIO*
6	*MIDRAT*	*MIDRAT*
7	ICANAD	SUJQOE
8	WIDZOA	FUDQIF
9	*QUJFUX*	*YAZYEF*
10	*BERFIP*	*QUJFUX*
11	*FAQVEA*	*BENXUP*
12	*XOVVIO*	*QUVDUH*
13	*QUVDUH*	WABTOK
14	REYCOP	TADCAE
15	*BENXUP*	*FAQVEA*

Table S7 DOI links of selected MOFs

MOF	DOI
AMUCOB	10.1039/c0ce00450b
SUHHOT	10.5517/ccrnvgp
XEHTUB	10.1007/s10904-012-9676-x
YAZYEF	10.1039/c2dt31090b
ICANEH	10.1039/c1ce05672g
MIDRAT	10.1021/cg0700317
ICANAD	10.1039/c1ce05672g
WIDZOA	10.1021/cg3014464
QUJFUX	10.1039/b909772d
BERFIP	10.1039/C3CE00049D
FAQVEA	10.1039/c1ce05829k
XOVVIO	10.1021/cg701164u
QUVDUH	10.1021/cg901012w
REYCOP	10.1039/c3ce26864k

BENXUP	10.1002/anie.201300821
MOGVAG	10.1016/j.jssc.2008.05.007
SUJQOE	10.1002/anie.200905898
FUDQIF	10.1002/ejic.200900144
WABTOK	10.1107/S1600536810048634
TADCAE	10.1039/c002573a

References:
(1) Todd, Michael . “Chapter 2: Minimum-Volume Ellipsoids.” *Minimum-Volume Ellipsoids*, Society for Industrial and Applied Mathematics, 2016, 11-23.
(2) Tang, D.; Gharagheizi, F.; Sholl, D. S., Adsorption-Based Separation of Near-Azeotropic Mixtures-A Challenging Example for High-Throughput Development of Adsorbents. *J Phys Chem B* **2021,** *125* (3), 926-936.
